# New year as a moment of change in pro-environmental product consumption: evaluating the habit discontinuity and self-activation hypotheses using a large UK retail dataset

**DOI:** 10.3389/fpsyg.2025.1550091

**Published:** 2025-04-23

**Authors:** Paul Haggar, Yasmin Sachdev, Lorraine Whitmarsh, James Goulding, Andrew Smith, Gavin Smith

**Affiliations:** ^1^School of Geography and Planning, Cardiff University, Cardiff, United Kingdom; ^2^Department of Psychology, University of Bath, Bath, United Kingdom; ^3^N/LAB, Nottingham Business School, University of Nottingham, Nottingham, United Kingdom

**Keywords:** pro-environmental consumption, temporal landmarks, habit discontinuity, value activation, fresh-start effect

## Abstract

**Introduction:**

The New Year, and the New Year’s Resolution tradition, may establish January as a moment of personal change: when there could be a temporal landmark for making a “fresh start,” a habit discontinuity, and value activation. As such, January may afford opportunities for personal pro-environmental lifestyle changes, such as by changing product choices.

**Method:**

To investigate this empirically, we analyzed existing data from a 2016 survey of retail customers (*N* = 12,968) linked to 35 months of their sales data (2012–2015) provided by a leading healthcare retailer in the United Kingdom. We compared sales in January to those in other months, focusing on sales of green product varieties and overall product sales (as a dematerialization indicator), and sales of two self-enhancing health product types (nicotine replacement therapy products and weight reduction products) for comparison.

**Results:**

Our results confirmed that sales of self-enhancing health products were greater in January than in other months, but we found limited evidence for pro-environmental consumption in January, and no evidence to support the habit discontinuity or value activation hypotheses.

**Discussion:**

We discuss these results with respect to behavior change intervention potential and moments of change theory.

## 1 Introduction

The consequences of global climate change – such as sea-level rise, extreme weather events, and depleted biodiversity – have widespread negative impacts upon human health and wellbeing, making mitigation of climate change a practical necessity (Lee et al., 2023). For individuals, behavioral and lifestyle change is of great importance in addressing this unprecedented challenge effectively (Whitmarsh et al., 2021). These changes include new beneficial consumption practices, whether adopting more environmentally sustainable technologies (e.g., electric vehicles adoption: Muratori et al., 2021), making product substitutions (e.g., eating less meat and more plant-based foods: Poore and Nemecek, 2018), or buying only what is necessary and no more (dematerializing: Whitmarsh et al., 2017). Innovative and effective policy is recommended to foster such changes, mindful that there may be contexts or situations that empower autonomous individual action (Verfuerth et al., 2023) and where behavior is more likely to change (Verplanken and Wood, 2006).

Shared culture is fundamental to shared social norms and goal pursuit (Heine and Ruby, 2010; Tomasello et al., 2005). In the United Kingdom and North America, January can be a time of change and renewal, whether this involves a general desire for self-change, or for self-reform (following the excess of the winter holidays). There is the formalized custom of explicitly making a commitment to change one’s behavior or attitude as a New Year’s Resolution (NYR). Psychologically, the New Year could constitute a “temporal landmark” (Peetz and Wilson, 2013; Shum, 1998) that guides memory and marks identity transition (Dai et al., 2014; Noble and Walker, 1997). For some, it can signal a “fresh start” (Beshears et al., 2021), a time to cease contemplating change and take action (Keller et al., 2019; Norcross et al., 2002). While January may not be the only significant temporal landmark (Dai et al., 2015; Gabarron et al., 2015; Peetz and Wilson, 2013), its cultural significance–for self-improvement and personal reflection–could make it suitable for encouraging natural and constructive behavioral change. Indeed, health behaviors are known to change at New Year; for example, gym memberships are far higher in January than any other month (Bloomberg, 2019). On the other hand, it is also possible that, for some, NYRs are not made in earnest, if at all, and January holds no special significance.

*Moments of change* occur when stable features of someone’s physical or social environment change (Thompson et al., 2011), and this can facilitate natural behavior change through habit discontinuity and value activation processes (Verplanken et al., 2008, 2018). Habits are behavioral propensities based in memory and they guide behavior automatically when cued by stable features in the environment with which they have become associated (Verplanken and Orbell, 2022). *Habit discontinuity* occurs when changes in the environment remove these cues, which then are not present to cue habitual responses, creating a window of opportunity within which people may find their actions do not occur in a fluid or effortless way but, at the same time, are enacted more deliberately, and with greater conscious control and intentionality (Verplanken and Wood, 2006). A different aspect of moments of change is that their disruptive character can lead to decisions being guided by core values, more than by transient or contingent considerations, and this is termed *value activation* (Verplanken et al., 2008; Verplanken and Holland, 2002). Hence, while habit discontinuity alone does not necessarily support the idea that moments of change lead to value-led behavior change, as opposed to self-interested or expedient behavior change, it supports this hypothesis alongside value activation. It is possible that the New Year is a moment of change. For instance, the holiday period prior to New Year may temporarily alter the physical or social environment, allowing change to be contemplated during the holidays and implemented afterwards.

At the same time, even if NYRs are made and taken seriously, then such resolutions to change ingrained behaviors are known for being unsuccessful. Such resolutions can inspire false hope for self-change including unrealistic expectations and assumptions (Polivy and Herman, 2002). Ingrained behaviors are likely habits, which are difficult to break while within the stable contexts that cue them (Wood et al., 2005). Hence the ‘sixth-of-January effect’ (NYR failure) may arise, where NYRs made during the winter holiday period fail and one falls back into one’s familiar routines, and the physical and social environment return to normal, in the New Year, triggering old habitual behavior patterns (Verplanken et al., 2018). This contrasts with more significant or disruptive moments of change (e.g., moving house, new parenthood) that may serve to weaken existing habits and may help one to maintain the resolve to change (Verplanken et al., 2008). Nevertheless, if around half of the UK population made NYRs with around 13% (potentially more in the future) making specifically pro-environmental NYRs (Nguyen, 2023; Fernandes, 2024), and others exploiting January as a temporal landmark, then there is a real possibility that even relatively small January-trends in pro-environmental behavior (such as in product choices) can be detected within larger datasets (Lavelle-Hill et al., 2020). The practical significance of this warrants investigation if, as one study indicated, January resolutions are more likely to be successful than resolutions during the rest of the year (Norcross et al., 2002).

In this article, we address three questions to evaluate the extent to which January (the beginning of the year) constitutes a natural moment of change.

1. Is self-improvement (e.g., health-focused) consumption greater in January than in other months?
2. Is pro-environmental consumption greater in January than in other months?
3. Is pro-environmental consumption more closely associated with environmental concern in January than in other months?

Self-improvement consumption was assessed through sales of two indicative health-product types, whereas pro-environmental consumption was assessed through sales of green products (to reflect pro-environmental product substitution) and through sales overall (to reflect dematerializing one’s consumption). While self-improvement through health-behavior change reflects traditional NYR goals, this study is (to our knowledge) the first study to consider whether pro-environmental NYR goals are detectable within sales data.

After reviewing relevant literature (Section 1.1) and stating our hypotheses (Section 1.2), we present our methods (Section 2), results (Section 3), and a discussion of our findings (Section 4).

### 1.1 Literature review

Periodic astronomical events guide the passage of time and structure our calendars (Considine and Considine, 1995). These events are cyclical: while the present, 1582 Gregorian, calendar dates each new year from January first, the date of a new year has historically differed (e.g., in the UK, to the start of Easter on Lady Day, March 25^th^), legally, religiously and by custom, and continues to differ for fiscal and academic purposes (Reingold and Derschowitz, 2001). The January-first new year is marked by the winter solstice (Aveni, 2002). New Year’s customs, or rituals, in the UK and North America blend different traditions: a Roman pagan tradition – where January hosted several rituals of physical and spiritual purification; the Celtic rituals of “wassail”; and Western Christian fasting and self-reflection observances (Aveni, 2002; George, 2020; Thorner, 1951). Though the exact origins of the New Year’s Resolution (NYR) are unclear, it is a secular ritual increasingly practiced across cultures (Hallinan et al., 2023).

Annual opinion polling surveys provide insight into the prevalence of NYRs and the types of resolutions being made. UK surveys in 2023 indicate that between 48 and 58% of respondents intended to make, or made, at least one NYR (Boyle and Pennarts, 2024; GoCompare, 2022), and 66% intended to make resolutions for 2024 (Boyle and Pennarts, 2024). Some survey results suggest that women may be somewhat more likely to report resolutions than men (62% versus 53% in 2023, and 55% versus 52% in 2022; Boyle and Pennarts, 2024). This UK polling data also shows indications of a rising 3-year trend in the proportion of British adults making NYRs each year; from 54% in 2022, to 58% in 2023, and to 66% in 2024 (Boyle and Pennarts, 2024). Such a rising trend could be age-related or generational, with older survey respondents, compared to younger ones, less frequently reporting NYRs (Boyle and Pennarts, 2024; Raven, 2022).

Of similar importance to the frequency of resolutions are the resolutions being made. Data from polling surveys also suggests common resolutions reflect financial goals (e.g., thrift), making time for family and friends, and one’s self-improvement and/or personal growth (Boyle and Pennarts, 2024; GoCompare, 2022). However, New Year’s resolutions centered around *health* are the most prevalent, including resolving to improve physical fitness, lose weight, and improve one’s diet (Boyle and Pennarts, 2024; GoCompare, 2022; Raven, 2022). This data is corroborated by the academic research available, where most resolutions concerned physical health, weight-loss goals, substance abstinence (e.g., from tobacco or alcohol), and finances (Marlatt and Kaplan, 1972; Moshontz de la Rocha, 2020). Similar patterns are also evident in online search engine search-term frequencies, which show, for instance, that searches using search terms such as “diet” and “healthy food” peak around January (Bayram and Ozturkcan, 2023; Cherchye et al., 2020; Dai et al., 2014). At the same time, it can be shown that gym visits and goal-commitments can increase immediately after the start of the New Year (Dai et al., 2014).

However, there is little evidence that NYRs reflect environmentalism or pro-environmental behavior change. Some evidence is available from social marketing campaigns encouraging annual abstentions, particularly Veganuary, which encourages veganism (i.e., animal-based product abstention) during January (Veganuary, 2022). Global participation in Veganuary has considerably increased in recent years, with approximately 25 million participants in 2024 (Veganuary, 2024). Given the sizeable carbon footprint associated with meat consumption (Ivanova et al., 2020), Veganuary participation – in some cases – amounts to being a pro-environmental NYR, though aimed at temporary rather than permanent dietary change. Furthermore, recent social media evidence shows environmentalist NYRs to be popular in parts of Europe (Hallinan et al., 2023), and polling data from 2023 shows 13% of British adults sampled resolved to become more conscious of their environmental impact (Nguyen, 2023), and 14% in 2024 (Fernandes, 2024). In light of mounting environmental concerns in the UK (Department for Business, Energy & Industrial Strategy, 2020; Department for Energy Security and Net Zero, 2024; Office for National Statistics [ONS], 2024), and the increasing number of British adults making New Year’s resolutions each year (Boyle and Pennarts, 2024; GoCompare, 2022), it is possible that pro-environmental lifestyle changes may increasingly propagate through individuals’ NYRs.

If so, then it is important to investigate NYRs to better understand whether they have the potential to encourage pro-environmental behavior change and so help address environmental issues such as global climate change. While self-improvement would seem, at first, to exclude environmental altruism, this is to neglect desires for intrinsic self-improvement (Geiger and Brick, 2023), which, like pro-environmental values (Steg et al., 2014), are associated with pro-environmental behavior (Unanue et al., 2016). With respect to green product choices, pro-environmental motivations are frequently identified as correlates through research (Sharma et al., 2023). Hence, if a substantial proportion of the UK population make pro-environmental NYRs (or otherwise form pro-environmental intentions in January) then some improvement in pro-environmental product purchasing should follow.

Academic research concerning NYRs is relatively scarce, and the studies in this area predominately use NYRs to investigate related phenomena, such as goal pursuit, self-regulation, and staged behavior change (e.g., Keller et al., 2019; Norcross et al., 2002; Oscarsson et al., 2020). One early study found that US college students’ weight-loss resolutions were unsuccessful in reducing their weight after 3-months, and 25% of other resolutions were also broken within a 3-month period (Marlatt and Kaplan, 1972). In the longer term, NYRs may be even less successful. One study of NYRs in Sweden found that, after 1 year, only 55% reported successfully sustaining their resolutions (Oscarsson et al., 2020). Another, longitudinal US, study found only 19% to be successful after 2 years and, among these, approximately 50% reported at least one ‘slip’ (Norcross and Vangarelli, 1988). However, their success relative to non-NYR change-resolutions is important to consider for comparison: one 6-month longitudinal study (Norcross et al., 2002) compared those with NYRs to ‘non-resolvers’ (those who contemplated change during the New Year and resolved to change soon afterwards but not in January) and those with NYRs reported greater goal success: a gap of 20% in the initial weeks, widening to 47% after a month, and remained wide at 42% after 6 months, with 46% of New Year resolvers successful compared to 4% of non-resolvers. It is important, however, to consider that, with some exceptions (e.g., Marlatt and Kaplan, 1972), behavior change is self-reported in these studies and, as such, may be subject to self-reporting biases, such as unwillingness in reporting resolution failure; more objective measures are preferable.

Trace evidence, such as records of product purchasing retained by retailers, provides an objective source of convergent evidence. However, research utilizing this approach to study New Year trends and resolutions is also scarce. In one study (Pope et al., 2014), healthy food shopping patterns were considered, using a participating sample’s purchases from 2010/11 to compare nutrition, calories, and food expenditure at different times of the year. They found that, in the post-holiday period (January to March), expenditure on nutritionally healthy foods increased by 29%, suggesting dietary behavior change following the holiday period (Thanksgiving to New Year’s). However, sales of unhealthy foods did not change between these seasons, indicating an overall increase in purchased calories (+9.3%). Calories in both seasons exceeded those in a comparison period (July to Thanksgiving) by 20% in January–March and 10% from Thanksgiving to New Year’s Day. Longitudinal evidence of changes in consumption as a result of the New Year was provided by Cherchye et al. (2020), who utilized 6 years of food purchasing data from 25,000 British households. They observed that the share of calorific expenditure of healthy goods decreased between January and December but–crucially–exhibited a ‘reset’ in January, a discrete increase in healthy food consumption. The authors postulate that this effect may be a result of New Year’s resolutions surrounding healthy diets, manifest in US and UK Google search trends (Cherchye et al., 2020; Dai et al., 2014, 2015), and supported by the aforementioned polling data (Boyle and Pennarts, 2024; GoCompare, 2022; Raven, 2022).

While reducing red meat consumption, and the carbon footprint of one’s diet more broadly, is an element in pro-environmental product consumption, a healthy diet is not necessarily an environmentally sustainable diet, and other forms of product consumption are also impactful. Presently, the only retail evidence surrounding the New Year concerns diets and/or healthy eating; while food product purchasing is a fundamental aspect of green consumption, due to the considerable contribution of the food sector to global GHGs (Poore and Nemecek, 2018) and the particularly detrimental carbon footprint of certain products, such as meat (González et al., 2020), the concept of green consumption is much wider (Peattie, 2010). Specifically, we consider green consumption in two ways. First, if consumers are environmentally motivated in their product choices, then we might expect them to substitute less for more environmentally sustainable products, such as those with natural ingredients or with smaller carbon footprints. Second, overall sales can reflect the materialization of consumption behavior (Whitmarsh et al., 2017): if a consumer tries to do more with less and make what they have last longer (dematerialize), then they are likely to also make fewer product purchases overall, even if they select from the same product varieties.

Despite the possibility of increasing environmental concern leading to increasingly pro-environmental NYRs, there are no studies to date, and to our knowledge, that have investigated pro-environmental consumption in the New Year specifically. Hence, our research contributes by (1) utilizing retail data to investigate green consumption in the New Year and (2) considering two different facets of green consumption (i.e., increased share of green product consumption and reduced overall product consumption).

### 1.2 Hypotheses

Our three research questions were expressed as six testable hypotheses. To address our first research question, whether self-improvement consumption is greater in January than in other months, we tested two hypotheses (H1 and H2).

*H1:* Nicotine Replacement (NR) products consumption is greater in January (than other months).

*H2:* Weight Reduction (WR) product consumption is greater in January (than other months).

To address our second research question, whether pro-environmental consumption is greater in January than in other months, we tested two further hypotheses (H3 and H4).

*H3:* Green Product (GP) sales are greater in January (than other months), allowing for all Green and Non-Green (GnG) product sales.

*H4:* Total Product (TP) sales are smaller in January (than other months).

To address our third research question, whether pro-environmental consumption is more closely associated with environmental concern in January than in other months, we tested two final hypotheses (H5 and H6).

*H5:* January Green Product (GP) choices (compared to other months) are explained by environmental concern, allowing for all Green and Non-Green (GnG) product sales.

*H6:* January Total Product (TP) sales (compared to other months) are explained by environmental concern.

We propose H1 and H2 on the basis that health goals such as these are most prevalent of NYRs (Boyle and Pennarts, 2024; GoCompare, 2022; Raven, 2022) and so provide an indication of changes one might detect due to known NYRs. We propose H3 and H4 for comparison; it is a possibility that pro-environmental change in January may be manifest in sales but not reflected in the NYRs reported in surveys. We propose H5 and H6 to consider the habit discontinuity and self-activation hypotheses together: if changes in sales in January are environmentally motivated, we would anticipate them to be most manifest in those concerned about the environment. Notably, in the absence of specific data on NYRs, we rely upon the month of January as a proxy. Also, we did *not* consider a symmetrical, fourth, research question, *is self-improvement consumption more closely associated with self-improvement motivation in January than in other months*? This is an important question, however we made use of an existing dataset which did not include an appropriate measure of self-improvement motivation. Another unavoidable limitation was that total product sales is not unambiguously linked with pro-environmental motivation but may be explained through economic motivation such as budgeting. Hence, New Year’s and making resolutions coincides with commercially important seasonal shopping events, such as the boxing-day or January sales in the UK, akin to “Black Friday” in the USA (Vu and Brinthaupt, 2018), during which shopping experiences, motivations and incentives can be atypical (Betts and McGoldrick, 1996). New Year’s may, equally, be a moment for financial economy following higher spending during the Christmas holidays.

## 2 Method

### 2.1 Data and design

We analyzed a survey dataset and a large retail dataset together. The survey had been conducted online between April and November 2016, sampling only loyalty card account customers from a leading retail pharmacy in the UK. Of 80,000 individuals contacted across the UK, 16,132 respondents gave their informed consent and participated with 12,968 completing the questionnaire. In addition to socio-demographic questions, the questionnaire also contained several psychographic questions. The large retail dataset contained records of shop-bought and online purchases from January 1st, 2012, through to November 30th, 2015. Unique loyalty account numbers of the respondents provided a key that we used to link these two datasets. In total, the survey participants spent approximately £24 million on 5 million products during these 35 months. In addition to sales, the retail dataset also included a list of almost all products sold at the shop, with short descriptive text for each, as well as typologies used by the retailer to classify products into broader product categories. Our secondary data analysis study, and the original questionnaire study for which the data was obtained (Lavelle-Hill et al., 2020), both received full ethical approval from the University of Nottingham.

We used the sales data to create four dependent variables and one control variable. Each was the number of unit sales of a broad product type. Self-improvement product sales were operationalized as nicotine replacement product sales and as weight loss product sales. Pro-environmental product sales were operationalized as green product sales and total product sales (where fewer sales in total were considered indicative of more dematerialized consumption). The sales of products from categories of product containing mixed green and non-green products were also used as a control variable in analyses addressing hypotheses H3 and H5.

These five variables were each implemented in two different ways. First, sales were aggregated to the calendar month level for each survey respondent. Hence, January sales were the mean average of sales made by each respondent in January 2012, January 2013, January 2014 and January 2015. (As December 2015 data was not available, December data takes the average of December in 2012, 2013, and 2014.) The varying levels of the 12 calendar months were used as a repeated measures categorical variable of ‘month’. As we were interested in the effect of January upon sales, we compared sales in January to sales in other months.

A second way in which the five dependent variables were implemented was with sales aggregated to the level of 4 three-month quarters for each respondent: July–September sales (Q3), October–December sales (Q4), January–March sales (Q1), and April–June sales (Q2). Hence, Q1 sales were the mean average of sales made by each respondent in January–March 2012, 2013, 2014 and 2015. Also, Q4 data takes the average of only October–December 2012, 2013, and 2014, due to missing Dec 2015 data. The four quarters were also used as a repeated measures variable, comparing sales in Q1 to sales in other quarters. The purpose of this second approach was to provide more conservative tests of H5 and H6 (three-months of sales are less likely to fluctuate than sales in a single month).

Different covariates in these ANCOVA design analyses were, variously: (1) mixed green and non-green product sales (GnG sales), (2) questionnaire-measured concern for the natural environment (environmental concern), and (3) questionnaire-measured socio-demographic variables, such as gender, age and household income. GnG product sales were used as a covariate in models of green product sales so as to statistically control for the propensity to buy nutrition and personal care products irrespective of whether or not they happen to be green varieties of these products. Environmental concern was used to better explain the motivation for purchasing green products (or buying fewer products). Analyses with and without socio-demographic covariates allowed us to consider the extent to which sales differences across time remained once person characteristics had been accounted for statistically. Measures used in this study are summarized in Appendix Table A1.

### 2.2 Product identification

Some actions are manifestly pro-environmental (e.g., activism, charitable donation). Others are less manifestly so, arising from mixed motivations (Whitmarsh, 2009). For the two health-improvement products we consider, deciding to buy them is likely a direct manifestation of desiring to quit smoking or lose weight (respectively) and so these products were identified directly from the retailer’s product typologies. Nicotine reduction products (*n* = 289) included nicotine replacement therapy products with different delivery systems: dermal (patches), oral (tablets, lozenges, gum, soluble strips), and inhalation (inhalators, nasal-sprays, and e-cigarettes). Weight loss products (*n* = 1,177) included: over-the-counter anti-obesity medications; weight-loss and exercise powders, shakes and supplements; wholefood snacks (e.g., dried fruits and nuts); some natural supplements and herbal teas (e.g., camomile); artificial sweeteners; diet-branded ready-meals and snacks; diet books; diet plans or packs; and body-weight measurement scales. There were 23.0 thousand sales of nicotine replacement products and 27.9 thousand sales of weight-loss products by the sample during the study period.

For pro-environmental product sales measurement operationalization, we assumed mixed motivations. We assumed an initial choice to buy a particular product type for a primary motivation (e.g., shampoo to wash hair) followed by a secondary choice to buy a variety of that product that meets one or more additional criteria (e.g., X brand shampoo because it smells nice), and targeted the second choice in measuring and analyzing green product choices. Thus, in identifying green products, we focused on identifying green varieties within the different product type categories used by the retailer. In this article, we use “green,” rather than “sustainable” or “pro-environmental,” in referring to these products to acknowledge that this perception is the customer’s and may not correspond to the objectively assessed environmental impact of the product so much as its appearance and what its manufacturers claim (Santa and Drews, 2023).

In identifying green products, we focused upon nutrition and personal-care products. The retailer’s product typologies included 148 nutrition and personal-care product types, each categorizing many individual product items. Of these, 52 types (35.1%) were screened due to either having relatively low-sales or low-variety. Of those that remained, 65 (43.9%) did not include a mixture of both green and non-green products. This left 31 types (20.9%) that were used to assess green product sales in this study (Table 1). Of these, green product varieties were identified using brands in 21 of these product types. All brands within a product type with above-average sales were qualitatively coded as either green or not-green. Green brands were defined as those marketed as either natural (natural ingredients claims, natural imagery) or environmentally sustainable (e.g., reduced packaging, reduced emissions, ecological preservation). Green brands were identified using archived contemporary customer-facing brand webpages (accessed using web.archive.org). By contrast, in the other 10 product types green product varieties were identified when they were described using indicative key-words in the short text descriptions recorded with them in the retailer’s data. For example, a “chicken sandwich,” in containing the word “chicken,” was likely a meat-based product and less environmentally sustainable than a non-meat alternative, so would be classified as a non-green product. Key words reflected either the intrinsic properties of being non-meat foods (5 nutrition product types), organic certified foods (4 nutrition product types – baby wet foods, baby snacks, groceries, and dairy) or comparatively reusable products (1 personal care product type - tampons). Key-words were identified by generating random lists of 100 descriptions and identifying key words from these until five randomly selected lists failed to identify any new key words. Table 1 summarizes information on sales of green products by type. For the green product sales variable used in analyses, sales of green products were aggregated across these product types.

**Table 1:** Green product type sales.

Order	Class	Type	Sales	% of Sales	Green Sales	% of Green Sales
1	N	Drinks	191,287	14.552	17,790	9.438
2	PC	Facial moisturisers	184,687	14.050	13,297	7.055
3	PC	Hair shampoo	111,917	8.514	1,903	1.009
4	N	Sandwiches	104,711	7.966	36,335	19.277
5	PC	Hair styling	101,670	7.735	915	0.485
6	PC	Shower gels	100,158	7.620	12,019	6.377
7	PC	Hair conditioner	90,251	6.866	4,874	2.586
8	PC	Body moisturisers	89,788	6.831	14,725	7.812
9	PC	Facial cleansers	44,070	3.353	6,258	3.320
10	PC	Hair colors	43,656	3.321	7,029	3.729
11	N	Baby wet foods	30,196	2.297	11,505	6.104
12	N	Salads	28,947	2.202	20,581	10.919
13	PC	Lip-salves/balms	24,532	1.866	1,447	0.768
14	PC	Tampons	23,694	1.803	118	0.063
15	PC	Wash accessories	18,517	1.409	2,129	1.130
16	PC	Facial scrubs	15,657	1.191	4,165	2.210
17	PC	Cos. applicators	15,468	1.177	2,552	1.354
18	PC	Eye MP remover	14,407	1.096	2,118	1.124
19	N	Baby snacks	13,982	1.064	8,921	4.733
20	PC	Eye gels/creams	12,697	0.966	1,752	0.930
21	N	Chilled drinks	12,456	0.948	4,796	2.544
22	PC	Body exfoliators	9,005	0.685	620	0.329
23	PC	Facial skin toners	8,971	0.682	2,090	1.109
24	PC	Face masks	8,881	0.676	4,805	2.549
25	PC	Pore cleansing	6,637	0.505	1,075	0.570
26	N	Savory foods	5,047	0.384	4,245	2.252
27	PC	Beauty foot care	1,677	0.128	2	0.001
28	N	Convenience food	608	0.046	346	0.184
29	N	Groceries	512	0.039	5	0.003
30	N	Dairy	263	0.020	22	0.012
31	N	Meat, fish and deli	114	0.009	50	0.026
	Total		1,314,463	100	188,488	100

“PC” is personal care product. “N” is nutrient product. “Eye MP Remover” is ‘eye make-up remover’. “Cos. Applicators” is ‘cosmetic applicators’. “Type” is product type. “Sales” column enumerates product sales; “Green Sales” column enumerates green product sales.

### 2.3 Analyses

Before analysis, 1,865 respondents (14.4%) were deleted listwise due to missing data. For our analyses, all continuous variables were then standardized. In models where all socio-demographic covariates were included, the Benjamini-Hochberg procedure was used (FDR = 10%) to allow for false discovery arising from multiple testing (Benjamini and Hochberg, 1995); results without this procedure are presented in the Supplementary Tables S5–S8.

Hypotheses were evaluated using multi-level (also known as mixed effect) linear regression models. These were implemented in R using the lme4 package (Bates et al., 2015), with the lmerTest package (Kuznetsova et al., 2017) to assess statistical significance using Satterthwaite’s method. Months (level 1) were nested in customers (level 2). Random intercepts were used in all models. In models in which environmental concern was used as a predictor, the random slope of environmental concern upon the dependent variable was also included, as this improved model fit. Diagnostic plots suggested heavy-tailed (leptokurtic) distributed level-1 residuals and some heteroscedasticity, however previous research shows linear mixed models (with larger samples at multiple levels) to be relatively robust to violations of level-1 normality and homoscedasticity assumptions (Luo et al., 2021; Schielzeth et al., 2020). While lme4 does not allow the direct specification of covariance structure, we found Lme4 results to be very similar to those obtained using a compound symmetry covariance structure implemented using the R package nlme (Pinheiro and Bates, 2000).

In Section 3.1 we consider descriptive statistics for the sample. In Section 3.2 we evaluate our first two hypotheses (H1 and H2), that sales of nicotine-replacement products and weight reduction products are greater in January than in other months, using two models (first and second models). In Section 3.3 we evaluate our third and fourth hypotheses (H3 and H4), that sales of green products are greater in January, and that sales of all products (in total) are less in January, than in other months, using two models (third and fourth models). Models one, two, three and four, in Sections 3.2 and 3.3, are models in which sales in each of 11 months (December is excluded due to limited degrees of freedom) are contrasted with the grand mean across all months (i.e., months are deviation coded); we obtained the same results with a single ‘January/Not-January’ binary variable. In Section 3.5 we evaluate our fifth hypotheses (H5), that green sales in January are explained by environmental concern, using two models (five and six). We then evaluate our sixth hypothesis (H6), that total product sales in January are explained by environmental concern, using two models (seven and eight). Models five and seven evaluate H5 and H6 (respectively) without sociodemographic covariates and models six and eight evaluate these hypotheses with these covariates. Section 3.5 also includes two additional exploratory analyses (models nine and ten). They pertain to H5 and H6 but consider groups of three calendar months (quarters). Quarters 4, 1 and 2 are compared to a reference period (quarter 3) using dummy coding. These analyses also go beyond our hypotheses by including all second-order interactions between quarters and environmental concern, to explore to what extent environmental concern may be involved in green consumption in more aggregated time-periods and in time-periods across the year rather than considering only the New Year.

## 3 Results

### 3.1 Sample

Table 2 shows that 91.1% of our participants were female, which is greater than in the UK population (Office for National Statistics, 2023c). Also, 47.9% of our sample have university-level education, which is a slightly higher proportion than the 39% in the UK population (Office for National Statistics, 2023a).

**Table 2:** Sample descriptive statistics (categorical variables).

Variable	Category	Frequency
Gender	Female	10,118 (91.1)
	Male (Ref)	985 (8.9)
Income	<£25,000	2,497 (22.5)
	£25,000–£34,999	1,833 (16.5)
	£35,000–£49,999	1,905 (17.2)
	£50,000–£99,999	2,263 (20.4)
	>£100,000	480 (4.3)
	Null (∅)	2,125 (19.1)
Education	School	540 (4.9)
	G.C.S.E.	2,318 (20.9)
	BTEC	627 (5.6)
	AS	141 (1.3)
	A-Level	2,164 (19.5)
	Bachelor’s	2,664 (24)
	Other Postgraduate	1,382 (12.4)
	Masters	940 (8.5)
	PhD	293 (2.6)
	Other advanced	34 (0.3)
Occupation	Unemployed/Sickness	492 (4.4)
	Semi/Unskilled manual	1,027 (9.2)
	Casual worker	2,492 (22.4)
	Skilled manual worker	3,047 (27.4)
	Supervisory	2,048 (18.4)
	Intermediate managerial	70 (0.6)
	Higher managerial	64 (0.6)
	Student	124 (1.1)
	Homemaker	1,299 (11.7)
	Retired/Semi-retired	211 (1.9)
	Full-time carer	96 (0.9)
	SME (Business Owner)	133 (1.2)
Region	Northern Ireland	490 (4.4)
	Wales	551 (5)
	Scotland	655 (5.9)
	Northwest	887 (8)
	Northeast	557 (5)
	Yorkshire (Ref)	1,313 (11.8)
	West Midlands	516 (4.6)
	East Midlands	788 (7.1)
	East Anglia	415 (3.7)
	London	462 (4.2)
	Southeast	978 (8.8)
	Southwest	437 (3.9)
	Null (∅)	3,054 (27.5)
Household Type	Living on my own	1,976 (17.8)
	Living on my own with children	312 (2.8)
	Living with partner (Ref)	4,757 (42.8)
	Living with partner and children	2,089 (18.8)
	Living with other adult family	1,717 (15.5)
	Living with other adult non-family	252 (2.3)
Marital Status	Single (never married)	1,870 (16.8)
	Married (Ref)	7,528 (67.8)
	Widowed	481 (4.3)
	Divorced	981 (8.8)
	Separated	199 (1.8)
	Null (∅)	44 (0.4)
Diet	Vegan or Vegetarian	1,021 (9.2)
	Other	10,082 (90.8)

“Null (∅)” refers to missing data or participants indicating they would prefer not to disclose this information. Reference categories used in inferential analyses are marked “Ref,” in parenthesis. For the Gender variable, Male was chosen as the reference value. Measures are summarized in Appendix Table A1.

Table 3 illustrates that the median age of our sample was 52, and the median income band was £25,000 to £34,999, both of which are comparable to the UK adult population (HM Revenue & Customs, 2024; Office for National Statistics, 2023b). Environmental concern is slightly less than we might expect (*M* = 4.77, *SD* = 1.37) based on representative UK opinion tracking survey results (Department for Business, Energy & Industrial Strategy, 2020; Department for Energy Security and Net Zero, 2024; Office for National Statistics, 2024). Table 3 also shows the average monthly sales for each product type, per capita; green products (*M* = 0.15, *SD* = 0.77), weight reduction products (*M* = 0.03, *SD* = 0.41), nicotine replacement products (*M* = 0.03, *SD* = 0.37), and total products (*M* = 6.93, *SD* = 8.35). As only approximately 13% of the UK adult population smoke cigarettes (Office for National Statistics, 2023d), nicotine replacement products are consequently relatively low. Likewise, though approximately 64% of the UK adult population is overweight or obese (Baker, 2023), only a certain proportion will seek to lose weight via weight reduction products (e.g., weight reduction medication or low-calorie diet supplements).

**Table 3:** Sample descriptive statistics (continuous variables).

Variable	*N*	*M* (*SD*)	*Mdn*	IQR
Age	11,103	50.59 (14.83)	52	24
Education (scale)	11,103	4.51 (2.33)	3	4
Income (scale)	11,103	2.1 (1.53)	2	2
Occupational SES (scale)	11,103	2.76 (1.47)	3	2
Environmental Concern	11,103	4.77 (1.37)	5	2
Green Sales	133,236	0.15 (0.77)	0	0
GnG Sales	133,236	1.56 (2.6)	1	2
Weight Reduction Sales	133,236	0.03 (0.41)	0	0
Nicotine Replacement Sales	133,236	0.03 (0.37)	0	0
Total Sales	133,236	6.93 (8.35)	5	7

“SES” is ‘socio-economic status’. “GnG Sales” are ‘green/not green sales’, i.e., all sales within the 31 product categories within which green products were identified. Measures are summarized in Appendix Table A1.

### 3.2 Self-improvement consumption greater in January than in other months

**Figure 1:**
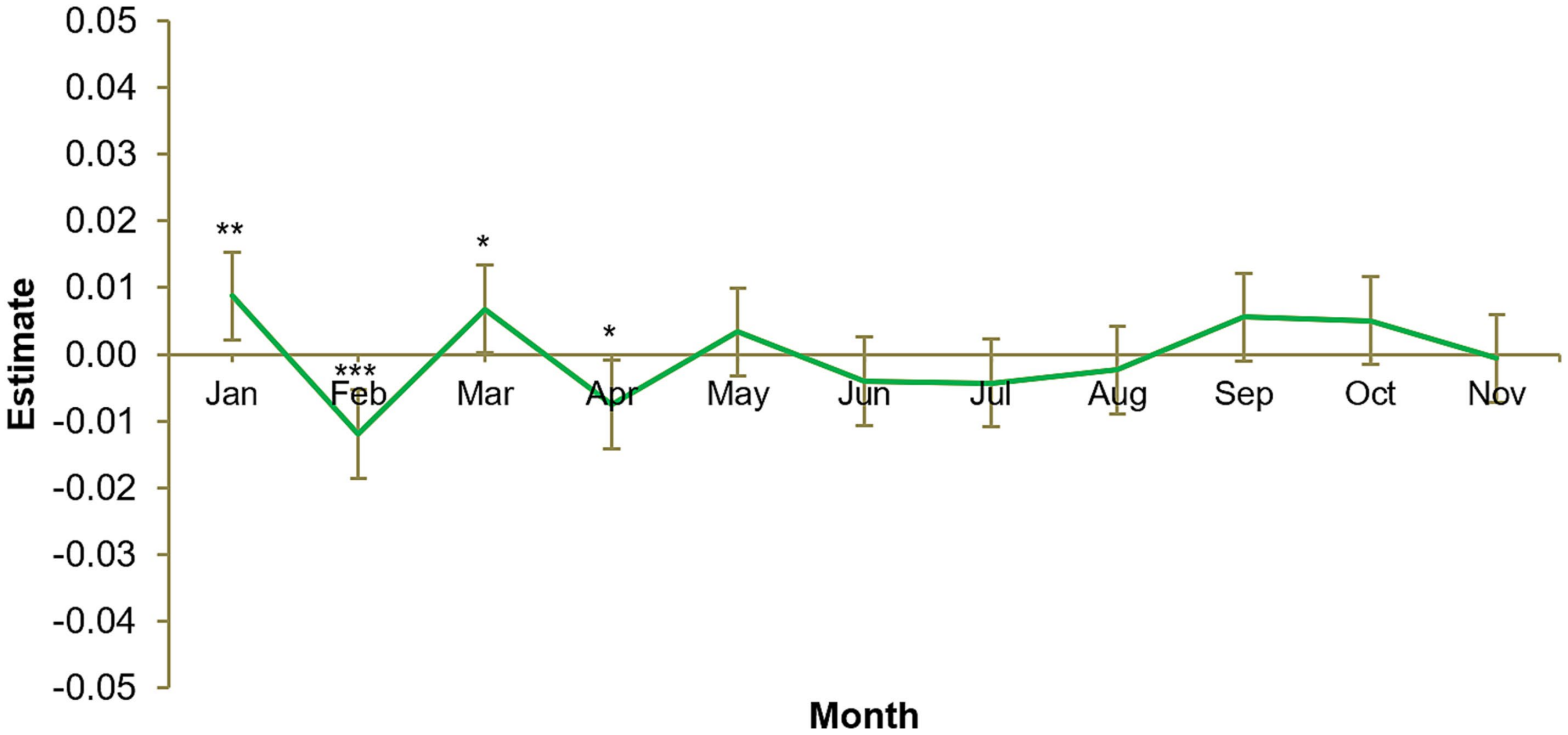
Monthly nicotine replacement product sales, January to November. Number of participants = 11,103, number of observations = 133,236. Each calendar month estimate is for the average of that month across 2012, 2013, 2014, and 2015. Each month is compared to the grand mean, i.e., time was deviation-coded and, hence, December was omitted to satisfy degrees of freedom. Error bars show 95% confidence interval. Full results available in Supplementary Table S1. *p < 0.05. **p < 0.01. ***p < 0.001.

Our first hypothesis was that nicotine replacement product consumption would be greater in January than in a typical month. Figure 1 illustrates the results of the first multi-level general linear regression model, in which nicotine replacement product sales are significantly greater in January (*B* = 0.01, *SE* = 0.003, *p* = 0.009), supporting this hypothesis. The chi-squared test of deviance showed the model to be statistically significant, χ^2^ (11) = 34.8, *p* < 0.001. Figure 1 also indicates that nicotine replacement product consumption is less than typical in February and April and greater than typical in March. This could be a dynamic effect due to (a) buying enough doses in 1 month for this month and the next and (b) some products in this category containing more than a month’s worth of doses.

**Figure 2:**
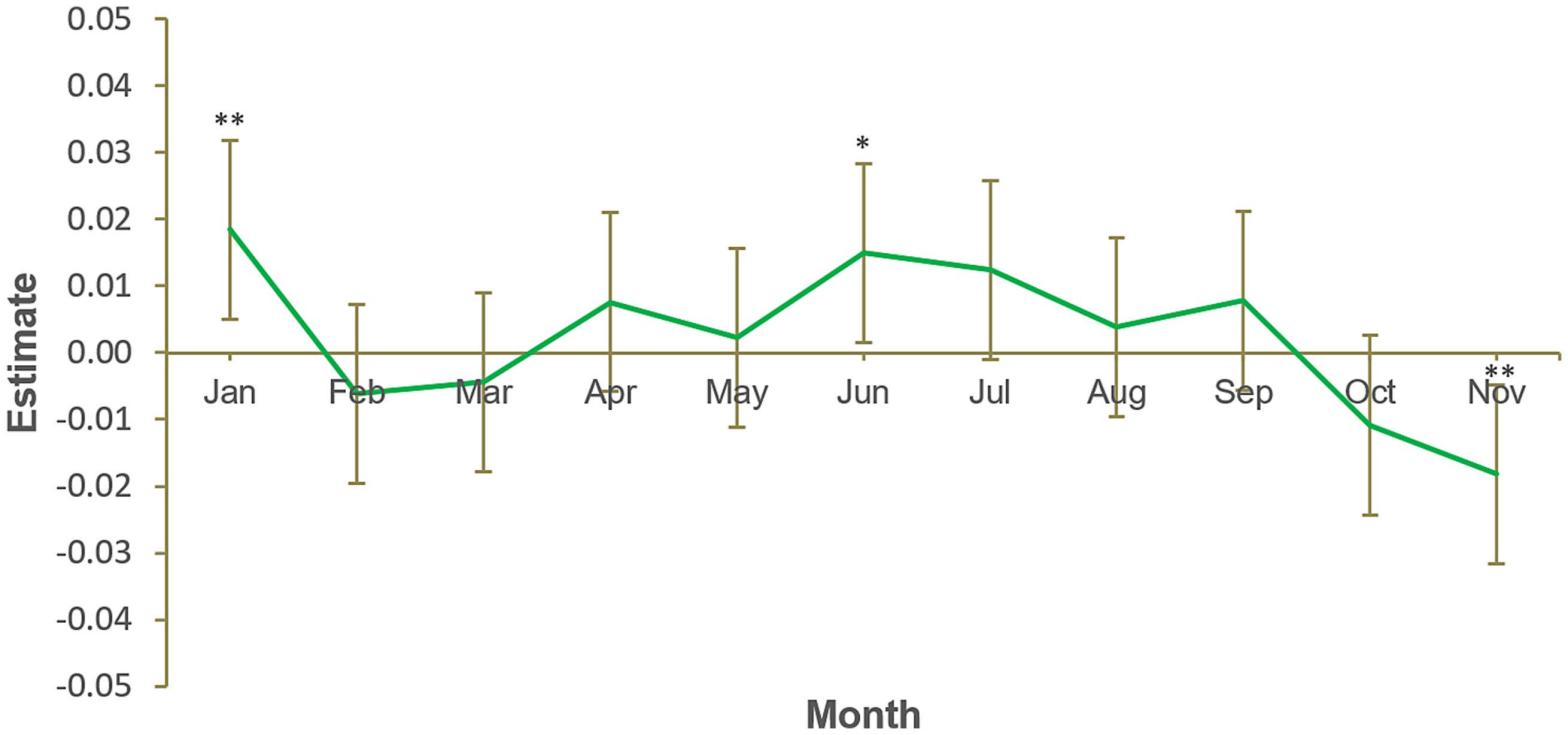
Monthly weight reduction product sales, January to November. Number of participants = 11,103, number of observations = 133,236. Each calendar month estimate is for the average of that month across 2012, 2013, 2014, and 2015. Each month is compared to the grand mean, i.e., time was deviation-coded and, hence, December was omitted to satisfy degrees of freedom. Error bars show 95% confidence interval. Full results available in Supplementary Table S2. *p < 0.05. **p < 0.01. ***p < 0.001.

Similarly, our second hypothesis, that weight reduction product consumption would be greater in January compared to a typical month, was also supported. The results of the second model (Figure 2) show that weight reduction product sales were significantly greater in January (*B* = 0.02, *SE* = 0.01, *p* = 0.007). The chi-squared test of deviance showed the model to be statistically significant, χ^2^(11) = 41.7, *p* < 0.001. Figure 2 also indicates that weight regulation product consumption is more than typical in June and less than typical in November. It is possible that diets are undertaken in June to improve one’s appearance for summer outdoor activities (e.g., the beach), while it is less likely that one would plan to diet during Christmas, when there may be social pressure to prepare and eat more food than usual.

### 3.3 Pro-environmental product consumption is smaller in January than in the average month

**Figure 3:**
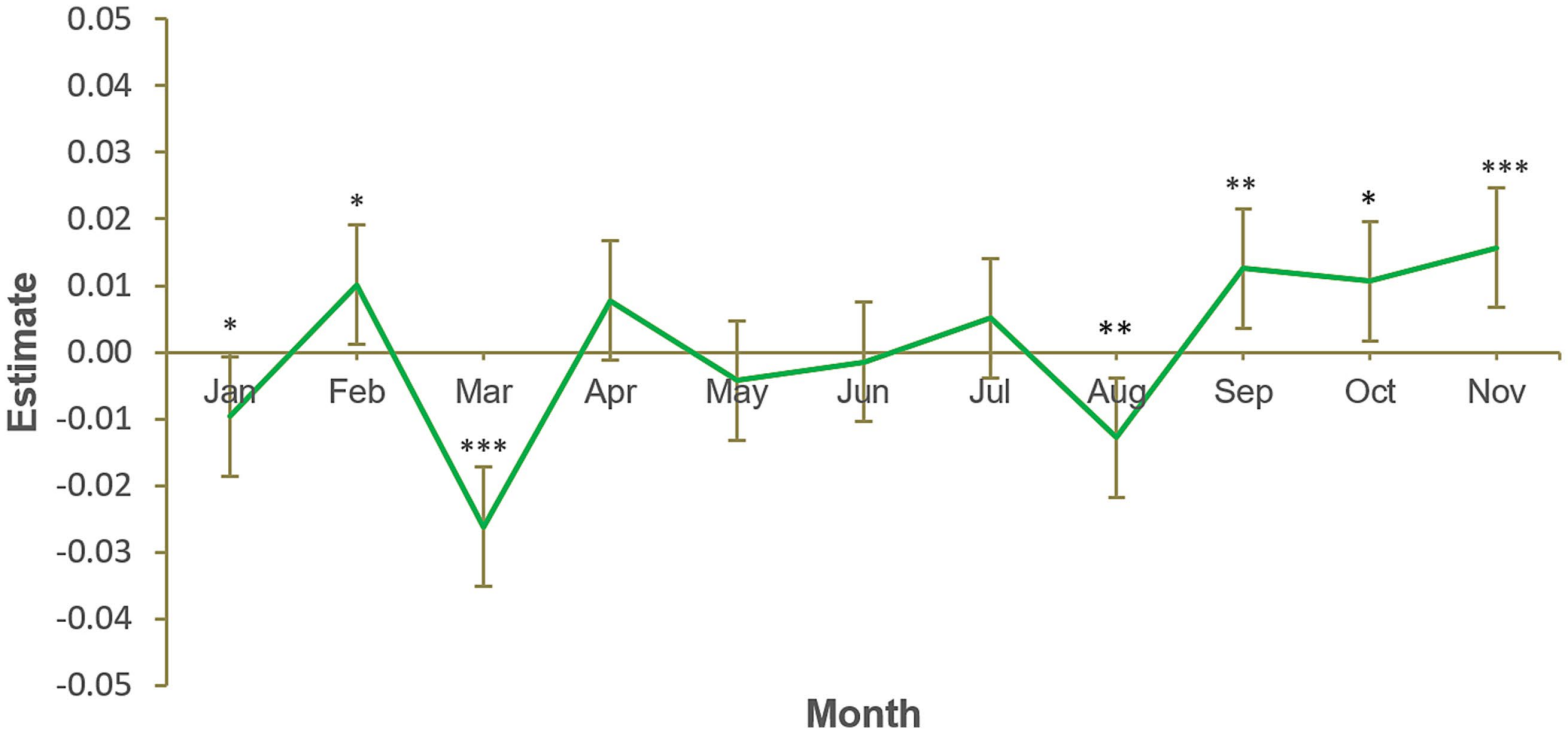
Monthly green product variety sales, January to November. Number of participants = 11,103, number of observations = 133,236. Each calendar month estimate is for the average of that month across 2012, 2013, 2014, and 2015. Each month is compared to the grand mean, i.e., time was deviation-coded and, hence, December was omitted to satisfy degrees of freedom. Error bars show 95% confidence interval. Full results available in Supplementary Table S3. *p < 0.05. **p < 0.01. ***p < 0.001.

Figure 3 illustrates the results of the third model, in which green product sales were significantly lower in January compared to a typical month (*B* = −0.01, *SE* = 0.005, *p* = 0.035). Hence, the third hypothesis, predicting that green product consumption would be greater in January when allowing for all GnG sales, was not supported. The chi-squared test of deviance showed this model to be statistically significant, χ^2^(12) = 36229.7, *p* < 0.001. Figure 3 also indicates that green product sales were more than typical in February, September, October and November, and less than typical in March and August. These differences are difficult to explain, however increased sales in winter months could be due to some green personal care products also tending to be premium products and, hence, more suitable as Christmas gifts.

### 3.4 Total product consumption in January is similar to average monthly sales

**Figure 4:**
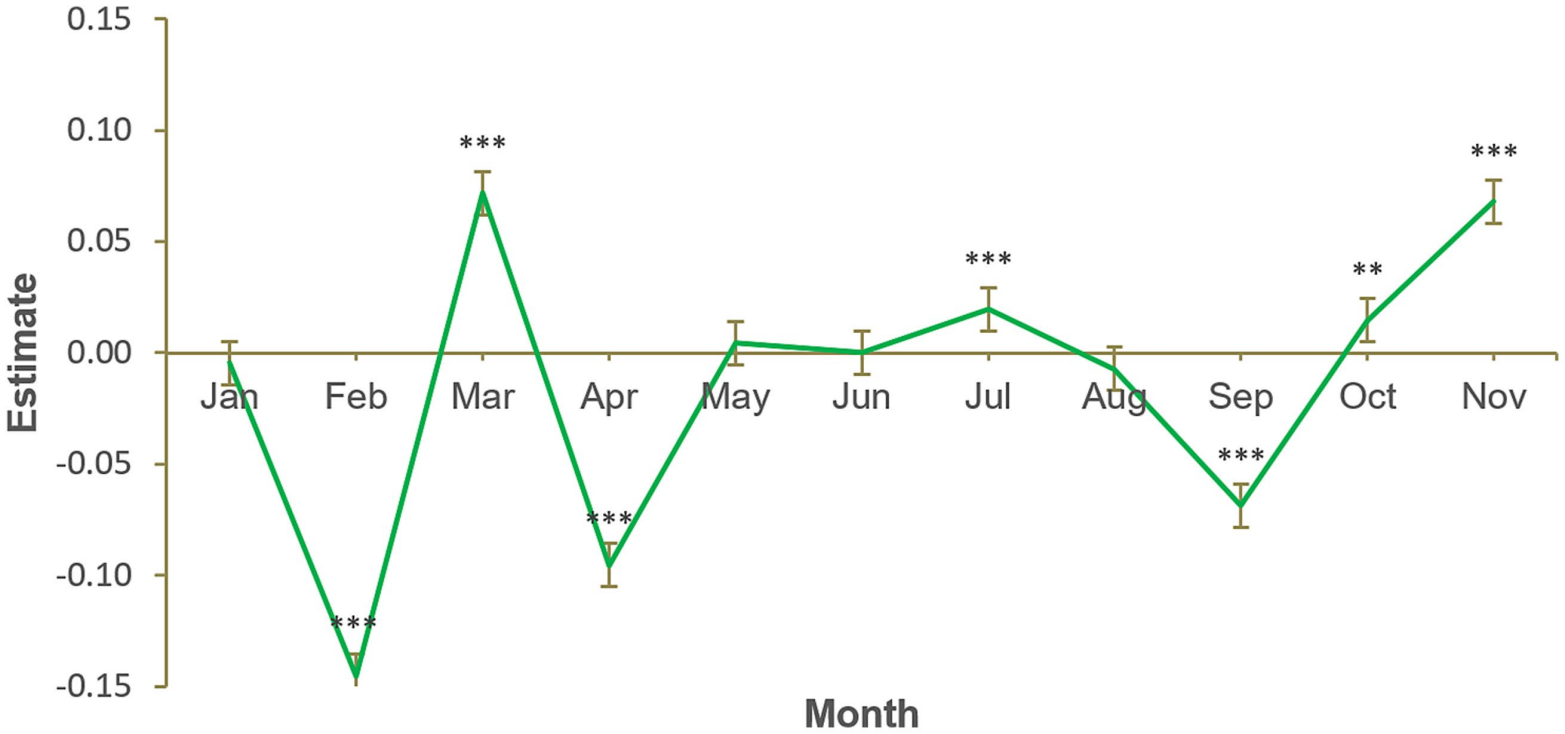
Monthly sales (all products), January to November. Number of participants = 11,103, number of observations = 133,236. Each calendar month estimate is for the average of that month across 2012, 2013, 2014, and 2015. Each month is compared to the grand mean, i.e., time was deviation-coded and, hence, December was omitted to satisfy degrees of freedom. Error bars show 95% confidence interval. Full results available in Supplementary Table S4. *p < 0.05. **p < 0.01. ***p < 0.001.

The results of the fourth model (Figure 4) do not support our fourth hypothesis, that total product sales would be significantly lower in January (*B* = −0.005, *SE* = 0.005, *p* = 0.335). Sales in January were closer to sales in an average month than were sales in February, April and September, which were lower. The chi-squared test of deviance showed this model to be statistically significant, χ^2^ (11) = 2449.3, *p* < 0.001. Figure 4 also indicates the total sales were less than typical in February, April and September and greater than typical in March, July, October and November. October and November sales can perhaps be attributed to Christmas purchases, whereas the pattern of increased March sales and reduced April sales could be related to Easter. Increased July sales could be related to summer products (e.g., sun protection).

### 3.5 Pro-environmental consumption was not more closely associated with environmental concern in January than in other months

We investigated whether pro-environmental product consumption was more closely related to environmental concern in January, compared to other months, via our fifth and sixth hypotheses. Table 4 presents first the results of the fifth model (without sociodemographic covariates) and the then the sixth model (with these covariates). The results indicate that environmentally concerned shoppers do purchase significantly more green products (*B* = 0.02, *SE* = 0.01, *p* < 0.001), but they do not do so more in January than the typical month (*B* = −3.33×10^−4^, *SE* = 0.005, *p* = 0.947). Hence, the fifth hypothesis, that green product choices in January are explained by environmental concern, when controlling for GnG sales, is not supported. These results remain the same when controlling for sociodemographic variables. The chi-squared tests of deviance showed the fifth model to be statistically significant, χ^2^ (2) = 36161.3, *p* < 0.001, and the sixth model to be statistically significant also, χ^2^ (38) = 36380.7, *p* < 0.001.

**Table 4:** Green product sales mixed effect models with and without socio-demographic covariates.

Effect	Estimate	*SE*	95% CI		*p*
			*LL*	*UL*	
Fixed effects					
Intercept	−0.003	0.01	−0.01	0.01	0.660
January	−0.01*	0.005	−0.02	−0.001	0.032
GnG Sales	0.52***	0.003	0.52	0.53	<2×10^-16^
Env. Concern	0.02**	0.01	0.01	0.03	0.001
Env. Concern x January	−3.33×10^-4^	0.005	−0.01	0.01	0.947

	Variance	*SD*	*r*
Random effects			
Individual	0.33	0.57	
Slope (Env.)	0.07	0.27	0.52
Residual	0.25	0.50	

Effect	Estimate	*SE*	95% CI		*p*
			*LL*	*UL*	
Fixed effects					
Intercept	0.30***	0.05	0.20	0.40	1.22 × 10^-9^
GnG Sales	0.52***	0.003	0.52	0.53	<2 × 10^-16^
Env. Concern	0.02**	0.01	0.01	0.03	0.003
Sociodemographic variables					
Age	−0.001**	0.001	−0.003	0.00	0.009
Gender	−0.21***	0.02	−0.25	−0.17	<2×10^-16^
Income	−0.03***	0.01	−0.04	−0.02	3.18×10^-7^
Income ∅	−0.07**	0.02	−0.11	−0.02	0.002
Household type					
Partner + Child	0.08***	0.02	0.05	0.12	1.91×10^-6^
Adult Non-Family	0.11*	0.05	0.02	0.20	0.018
Veg. Diet	0.09***	0.02	0.05	0.13	4.32×10^-5^

	Variance	*SD*	*r*
Random effects			
Individual	0.32	0.57	
Slope (Env.)	0.07	0.27	0.53
Residual	0.25	0.50	

CI = confidence interval; LL = lower limit; UL = upper limit. GnG Sales = total sales of products for all product types containing some green products. Number of participants = 11,103, number of observations = 133,236. In the model with socio-demographic covariates, the Benjamini Hochberg procedure (FDR = 10%) was applied to select significant predictors; a full model is provided in the Supplementary Table S5. Variables followed by “(∅)” refer to missing data or participants indicating they would prefer not to disclose this information. For the Gender variable, Male was chosen as the reference value. *p < 0.05. **p < 0.01. ***p < 0.001.

Similarly, the sixth hypothesis, that total product sales in January would be explained by environmental concern, was not supported. Table 5 presents the results of the seventh model (without sociodemographic covariates) and then the eighth model (with these covariates). The results indicate that there was no main effect of environmental concern on total product sales (*B* = 0.003, *SE* = 0.01, *p* = 0.739), nor was environmental concern significantly associated with total sales in January (*B* = −0.01, *SE* = 0.01, *p* = 0.058). These results remain the same when controlling for sociodemographic variables. The chi-squared test of deviance showed the seventh model to be statistically significant, χ^2^ (1) = 4.5, *p* = 0.034, and the eighth also, χ^2^ (37) = 643.6, *p* < 0.001.

**Table 5:** Total product sales mixed effect models with and without socio-demographic covariates.

Effect	Estimate	*SE*	95% CI		*p*
			*LL*	*UL*	
Fixed effects					
Intercept	0.001	0.01	−0.01	0.02	0.867
January	−0.01	0.01	−0.02	0.01	0.340
Env. Concern	0.003	0.01	−0.01	0.02	0.739
Env. Concern x January	−0.01	0.01	−0.02	0.00	0.058

	Variance	*SD*	*r*
Random effects			
Individual	0.67	0.82	
Slope (Env.)	0.03	0.17	0.11
Residual	0.30	0.55	

Effect	Estimate	*SE*	95% CI		*p*
			*LL*	*UL*	
Fixed effects					
Intercept	−0.17*	0.07	−0.30	−0.04	0.013
Sociodemographic variables					
Age	−0.01***	0.001	−0.01	−0.01	<2×10^-16^
Gender	0.18***	0.03	0.12	0.23	5.20×10^-10^
Income	0.08***	0.01	0.06	0.10	<2×10^-16^
Income ∅	0.15***	0.03	0.09	0.21	3.84×10^-7^
SES	0.04***	0.01	0.02	0.06	1.06×10^-4^
Occupation					
Student	−0.27*	0.11	−0.48	−0.06	0.011
Household type					
Parent + Child	0.11***	0.02	0.06	0.16	5.49×10^-6^

	Variance	*SD*	*r*
Random effects			
Individual	0.62	0.79	
Slope (Env.)	0.03	0.18	0.13
Residual	0.30	0.55	

CI = confidence interval; LL = lower limit; UL = upper limit. Number of participants = 11,103, number of observations = 133,236. In the model with socio-demographic covariates, the Benjamini Hochberg procedure (FDR = 10%) was applied to select significant predictors; a full model is provided in the Supplementary Table S6. Variables followed by “(∅)” refer to missing data or participants indicating they would prefer not to disclose this information. For the Gender variable, Male was chosen as the reference value. *p < 0.05. **p < 0.01. ***p < 0.001.

### 3.6 Exploratory analyses: quarterly sales

**Figure 5:**
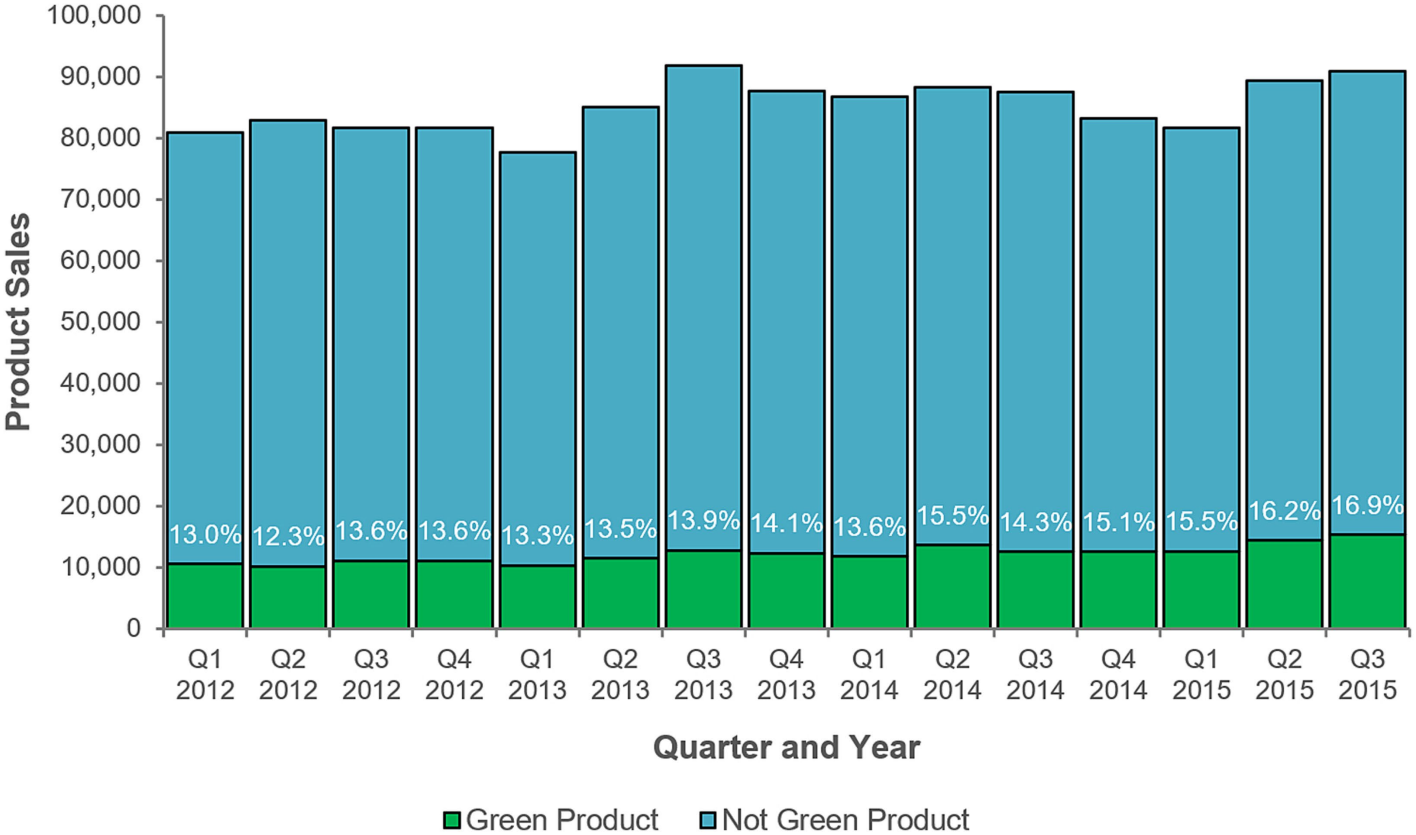
Quarterly green product sales, 2012–2015. “Not Green Product” indicates products of the same types as green products but that are not green varieties. Q1 = January, February, and March. Q2 = April, May, and June. Q3 = July, August and September. Q4 = October, November, and December. Q4 2015 sales data were incomplete.

**Figure 6:**
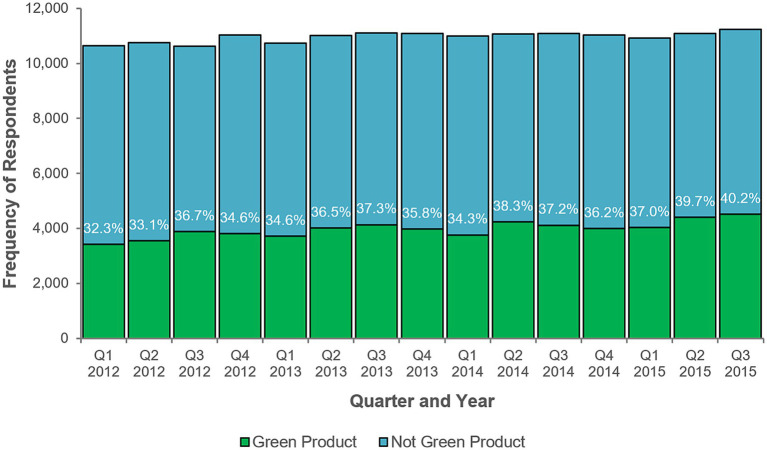
Quarterly frequencies of green product purchasers. Frequencies are of respondents who are recorded as buying at least one unit of the product during the time period. “Not Green Product” indicates products of the same types as green products but that are not green varieties. Q1 = January, February and March. Q2 = April, May, and June. Q3 = July, August, and September. Q4 = October, November, and December. Q4 2015 sales data were incomplete.

Some exploratory analyses were made using quarterly time-periods instead of monthly time-periods. Figure 5 shows sales of green products in the sample across all quarters, and Figure 6 shows the frequency of buyers of green products in the sample across all quarters. Each suggests a trend whereby green product variety sales (i.e., as a percentage of those types of products where green varieties were available), and those who buy green products, increased over time. As a descriptive finding from exploratory analysis, this is a tentative conclusion subject to further empirical verification.

Subsequent exploratory analyses using quarterly time periods offered no additional support for the fifth and sixth hypotheses. Table 6 shows the results of the ninth model; product consumption was significantly greater in the first (*B* = 0.01, *SE* = 0.004, *p* < 0.001) and second (*B* = 0.02, *SE* = 0.004, *p* < 0.001) quarters, and sales were greater for more environmentally concerned individuals (*B* = 0.02, *SE* = 0.01, *p* < 0.001). The chi-squared test of deviance showed this model to be statistically significant, χ^2^ (11) = 178017.2, *p* < 0.001.

**Table 6:** Quarterly green product sales mixed effect model with socio-demographic covariates.

Effect	Estimate	*SE*	95% CI		*p*
			*LL*	*UL*	
Fixed effects					
Intercept	0.14***	0.03	0.10	0.19	9.07 × 10^−9^
GnG sales	0.70***	0.002	0.69	0.70	<2 × 10^−16^
First quarter	0.01**	0.004	0.003	0.02	0.009
Second quarter	0.02***	0.004	0.02	0.03	1.37 × 10^−7^
Env. concern	0.02***	0.01	0.01	0.04	7.95 × 10^−5^
Sociodemographic variables					
Age	−0.02**	0.01	−0.04	−0.01	0.001
Gender	−0.08***	0.02	−0.21	−0.14	4.21×10^−20^
Income	−0.05***	0.01	−0.07	−0.03	7.18×10^−9^
Income ∅	−0.07**	0.02	−0.11	−0.03	0.001
Household type					
Partner + Child	0.08***	0.02	0.05	0.11	1.66×10^−6^
Adult non-family	0.11*	0.04	0.03	0.19	0.011
Veg. diet	0.10***	0.02	0.06	0.14	8.64×10^−8^

	Variance	*SD*
Random effects		
Individual	0.28	0.53
Residual	0.34	0.58

CI = confidence interval; LL = lower limit; UL = upper limit. GnG Sales = total sales of products for all product types containing some green products. Number of participants = 11,103, number of observations = 133,236. The Benjamini Hochberg procedure (FDR = 10%) was applied to select significant predictors; a full model is provided in the Supplementary Table S7. For the Gender variable, Male was chosen as the reference value. *p < 0.05. **p < 0.01. ***p < 0.001.

Table 7 shows the results of the tenth model. Here, total product sales were significantly greater in the fourth (*B* = 0.08, *SE* = 0.005, *p* < 0.001) and first (*B* = 0.01, *SE* = 0.005, *p* = 0.018) quarters. While total product sales generally increase in the fourth quarter (*B* = 0.08, *SE* = 0.005, *p* < 0.001), the increase is smaller amongst environmentally concerned individuals. (*B* = −0.01, *SE* = 0.005, *p* = 0.034), perhaps due to pro-environmentally-motivated dematerialization in October, November and December. The chi-squared test of deviance showed this model to be statistically significant, χ^2^ (11) = 102072.1, *p* < 0.001.

**Table 7:** Quarterly total product sales mixed effect model with socio-demographic covariates.

Effect	Estimate	*SE*	95% CI		*p*
			*LL*	*UL*	
Fixed effects					
Intercept	−0.20***	0.04	−0.27	−0.13	1.53 × 10^−8^
Fourth quarter	0.08***	0.005	0.07	0.09	<2 × 10^−16^
First quarter	0.01*	0.005	0.002	0.02	0.018
Env. Concern x Fourth quarter	−0.01*	0.005	−0.02	−0.001	0.034
Sociodemographic variables					
Age	−0.09***	0.01	−0.11	−0.07	1.74×10^−17^
Gender	0.17***	0.03	0.12	0.22	3.04×10^−10^
Income	0.11***	0.01	0.09	0.14	1.97×10^−20^
Income ∅	0.15***	0.03	0.09	0.20	3.18×10^−7^
SES	0.05***	0.01	0.03	0.08	1.26×10^−4^
Occupation					
Student	−0.25*	0.10	−0.44	−0.05	0.014
Retired	0.09*	0.04	0.01	0.17	0.021
Region					
E. Midlands	−0.08*	0.04	−0.15	−0.01	0.030
N.W. England	−0.08*	0.03	−0.14	−0.01	0.025
Household type					
Partner + Child	0.10***	0.02	0.06	0.15	4.42×10^−6^
Veg. diet	0.06*	0.03	0.01	0.12	0.014

	Variance	*SD*
Random effects		
Individual	0.57	0.76
Residual	0.41	0.64

CI = confidence interval; LL = lower limit; UL = upper limit. Number of participants = 11,103, number of observations = 133,236. The Benjamini Hochberg procedure (FDR = 10%) was applied to select significant predictors; a full model is provided in the Supplementary Table S8. For the Gender variable, Male was chosen as the reference value. *p < 0.05. **p < 0.01. ***p < 0.001.

## 4 Discussion

Lifestyle change is required to address the threat that global climate change poses to human wellbeing and the natural environment (Lee et al., 2023; Whitmarsh et al., 2021). Consumption behavior is an important aspect of this, to the extent that primary resource production and goods manufacturing each make substantial contributions to greenhouse gas emissions (Ivanova et al., 2020). Timing is of importance when developing interventions and policies to encourage pro-environmental behavior change, because there are likely moments of change during which people may be more receptive to these efforts and may also be more inclined to engage in self-directed attempts to change their own behavior (Thompson et al., 2011; Verplanken et al., 2018; Verplanken and Wood, 2006).

In this study, we evaluated the New Year (the month of January) as such a moment. There is some supporting evidence for this idea. Representative UK polling shows around half the UK population report New Year’s Resolutions (Boyle and Pennarts, 2024). Although NYRs have a low success rate (Keller et al., 2019; Marlatt and Kaplan, 1972; Norcross et al., 2002; Norcross and Vangarelli, 1988; Oscarsson et al., 2020), they can show a superior success rate to other change resolutions (Norcross et al., 2002). Therefore, we might expect to see some evidence of these successful changes reflected in consumer product choices, at least in larger samples. It has been theorized that January offers a temporal landmark for making a fresh start, and several studies provide support for the effectiveness of temporal landmarks in facilitating personal behavior change (e.g., Dai et al., 2015; Gabarron et al., 2015; Peetz and Wilson, 2013). It is also possible that the preceding winter holidays, with the disruption to regular routines and social situations, lead to a habit discontinuity and may also activate core values, allowing these to guide choices in the New Year (Verplanken et al., 2008). In the present study, we addressed this question through an analysis of nearly 5 years of purchasing data from a leading UK retailer linked to data from a questionnaire completed by loyalty card customers.

Quitting smoking and losing weight are common NYRs (Raven, 2022). Our results show that sales of nicotine replacement products and of weight loss products are each significantly greater in January compared to sales in a typical month, which is consistent with NYR-based purchasing during January. These findings provide circumstantial evidence for the effectiveness of NYRs in inspiring behavior change attempts that have real impacts on consumption choices, with the implication that, should pro-environmental behaviors become popular NYRs, similar pro-environmental consumption choices could be made. Our results add value in so far as retailer sales records, as a source of objective information, tend to rule-out self-report biases such as socially desirable responding or failures of memory and, unlike internet search engine search term frequencies, correspond to actions taken more than actions contemplated.

However, we found few indications in the literature that pro-environmental behavior change was a frequent choice of NYR and, consistent with this, our comparison results from green product sales and total sales were mixed. Inconsistent with pro-environmental consumer dematerialization, total product sales in January were typical. Similarly, and contrary to our hypothesis that consumers would buy *more* green products in the New Year, we found fewer green product sales in January than in a typical month (once allowing for the overall sales of personal care products and nutrition products). In the absence of strong pro-environmental motivations, these findings may be more consistent with household economizing. Total sales in *February* were the lowest of any month, so it is possible that total sales in both *January* and *February* are lower due to post-Christmas economizing, but that sales in January are offset by greater sales-volumes from the January sales (Vu and Brinthaupt, 2018). Second, green products are often sold at a price-premium, and so it is possible that the fewer purchases of these in January can be partly attributed to consumers temporarily switching to budget varieties to save money at this time of year.

It can be difficult to differentiate economic and value-based motivations from green product sales alone, hence, our research also considered the possibility that differences in pro-environmental sales in January would be dependent upon an individual’s degree of environmental concern, however we found no clear evidence to support this. Our findings showed that environmentally concerned individuals purchased more green products in a month, consistent with environmentally concerned individuals valuing the pro-environmental characteristics of these products. However, we found no evidence that environmentally concerned individuals purchased any more green products in January (or any fewer products in total) in January, compared to their purchasing in an average month. We obtained the same result with or without taking socio-demographic differences in sales into account, and when the New Year was operationalized as the first three-months of the year rather than January by itself. These results are inconsistent with the New Year as a moment of change where habit discontinuity and value-activation facilitate the adoption of more environmentally sustainable consumer behavior patterns or where the New Year represents a “fresh start” opportunity for adopting more sustainable consumer lifestyles.

Comparing these results to those obtained for self-improving health behaviors, it is appropriate to conclude that the New Year is a time during the year when individuals resolve to improve themselves, such as by adopting healthier behavior patterns, but that such resolutions have not extended, in the recent past, to adopting more environmentally sustainable consumer behavior, irrespective of the level of concern one has for the environment. However, it is also important to qualify this result by considering several limitations of our study.

The first limitation is that our findings were from a single large retailer, so (a) may reflect only the mass market and not the niche markets within which green products may be mostly traded (Wood et al., 2018), (b) we were not able to measure NYRs directly, but only by-proxy by comparing time-periods, which would make a causal link more difficult to detect, and (c) the demographic composition of our sample, e.g., 91% female and 48% university-educated, diverges from that of the general population, hence results may not be generally applicable. Hence, a study design measuring, manipulating or sampling-for NYR-holding, and considering sales in niche markets or sales across many retailers, could potentially make the effect of NYRs easier to isolate methodologically. Also, in using secondary data, where self-improvement motivations (e.g., relating to personal health) were not measured, we were unable to test the extent to which the greater January levels of sales-improvement product sales could be linked to habit discontinuity and/or value activation, and it remains a possibility that other seasonal variations may ultimately account for the sales patterns we show here. A fine-grain analysis within January could also offer an opportunity for detecting short-lived responses earlier in the month. Finally, our identification of green product varieties was made through marketing claims of manufacturers rather than through objective means, which means we cannot guarantee either that measured green product sales are more sustainable than other sales or that pro-environmental customers were exposed to, or believed, these marketing claims. Moreover, in identifying green products through claims of natural composition or environmentally sustainable manufacture, our measurement does not reflect economic, cultural or social aspects of sustainability.

Nevertheless, our results tend to suggest that NYRs are not presently suitable moments for value-motivated product-choice changes, which is of practical importance, given that much past research has considered value activation and habit discontinuity with respect to moving house, as a moment of change, and/or transportation choices, as environmentally significant behaviors (e.g., Haggar et al., 2019; Thomas et al., 2016; Verplanken et al., 2008; Verplanken and Roy, 2016; Walker et al., 2015). If these effects cannot be replicated across a wide range of events and behavioral domains, then this limits the generalizability of these broader hypotheses, and raises the prospect that effects noted within particular contexts or situations may be found to have more mundane explanations, such as the changing balance of practical advantages and disadvantages.

More generally, our study considered only naturally occurring events and so is limited in its implications concerning behavior change interventions. For instance, an important question that future research might clarify is the question of whether NYRs (due to their roots in a particular tradition) may tend to cover certain types of resolutions, these reflecting mostly self-enhancing values, rather than more altruistic ones, making it no easier to persuade or nudge individuals to make altruistic resolutions like environmental protection in the New Year. It is also by no means certain, beyond a minority who take them seriously, that NYRs are sufficiently culturally ingrained to form the basis for widespread behavior change in the population. However, it is important to consider the impact of digital culture and generational value change potentially accelerating cultural change and proliferation around NYRs (Hallinan et al., 2023). Likewise, in studying the potential of NYRs for behavior change, the timing of interventions is of particular interest to consider (Schäfer et al., 2012; Thomas et al., 2016), because the decision to change may be made in advance of the moment of change when it is implemented (Haggar et al., 2019). This is also consistent with the idea of the New Year as a temporal landmark for implementing changes, rather than of starting to plan or formulate future change (Dai et al., 2014, 2015). Similarly, closer further study to differentiate between different theoretical accounts (e.g., temporal landmarks, habit discontinuity) would be beneficial. One avenue would be to consider different graduations of temporal change decomposed using timeseries, so that (for instance) annual differences could be separated from annual cycles.

Our exploratory analyses showed that, contrary to overall greater sales before Christmas, environmentally concerned shoppers made fewer purchases in the final quarter of the year (October, November, and December) than shoppers less environmentally concerned. We did not hypothesize this outcome and hence it is subject to future confirmation. However, a tentative explanation may be found in environmental concern manifesting in reduced consumption during the festive period, such as purchasing fewer ‘disposable’ gift items. This is consistent with Christmas as traditionally a time of charity and altruism, which may manifest in greater consideration of environmental issues for those most environmentally concerned. Hence, future research in this field could consider the way in which environmentally motivated households might perceive Christmas as an opportunity for reducing excessive material consumption and whether this might be a useful moment to target with sustainable behavior change interventions.

In conclusion, our study of the calendar-month product sales of loyalty-card customers at a leading healthcare retailer in the United Kingdom found evidence that self-improving health-product sales (nicotine replacement and weight-loss products) were greater in January than in an average month, but did not find clear evidence that environmentally concerned customers purchased more green product varieties in January or purchased fewer products in January (which would have been indicative of dematerialized shopping). These results are valuable, both in confirming the potential of the New Year (or New Year’s Resolutions) as a moment that can encourage self-improving consumer choices but also in confirming that these are less likely to extend to the pursuit of pro-environmental behavioral goals that affect product choices. However, concerns about environmental issues such as climate change are mounting, and it remains possible that adopting more pro-environmental lifestyles may become a popular New Year’s Resolution in the future.

## Data Availability

The dataset presented in this article is proprietary and not readily available. Requests to access these datasets should be directed to James Goulding, James.Goulding@nottingham.ac.uk.
