# Supplementary material for: New year as a moment of change in pro-environmental product consumption: evaluating the habit discontinuity and self-activation hypotheses using a large UK retail dataset
Source: Front Psychol. 2025 Apr 23;16:1550091. doi: 10.3389/fpsyg.2025.1550091 (PMC12055770; doi:10.3389/fpsyg.2025.1550091)
Supplement: Supplementary file 1 [file Supplementary_file_1.docx]

**Supplement**

**Table S1**

*Monthly nicotine replacement product sales, January to November.*

| Effect | Estimate | *SE* | 95% CI | | *p* |
| --- | --- | --- | --- | --- | --- |
|  |  |  | *LL* | *UL* |  |
| Fixed effects |  |  |  |  |  |
| Intercept | -4.07x10^-13^ | .01 | -.02 | .02 | 1.00 |
| January | .01** | .003 | .002 | .02 | .009 |
| February | -.01*** | .003 | -.02 | -.01 | 4.04x10^-4^ |
| March | .01* | .003 | .00 | .01 | .044 |
| April | -.01* | .003 | -.01 | -.001 | .026 |
| May | .003 | .003 | -.003 | .01 | .321 |
| June | -.004 | .003 | -.01 | .003 | .230 |
| July | -.004 | .003 | -.01 | .002 | .203 |
| August | -.002 | .003 | -.01 | .004 | .491 |
| September | .01 | .003 | -.001 | .01 | .099 |
| October | .01 | .003 | -.002 | .01 | .133 |
| November | -.001 | .003 | -.01 | .006 | .860 |
|  | Variance | *SD* |  |  |  |
| Random effects |  |  |  |  |  |
| Individual | .86 | .93 |  |  |  |
| Residual | .14 | .37 |  |  |  |

*Note.* CI = confidence interval; *LL* = lower limit; *UL* = upper limit. Number of participants = 11,103, number of observations = 133,236. Each calendar month estimate is for the average of that month across 2012, 2013, 2014, and 2015. Each month is compared to the grand mean, i.e. time was deviation-coded.

** p* < .05. ** *p* < .01. *** *p* < .001.

**Table S2**

*Monthly weight reduction product sales, January to November.*

| Effect | Estimate | *SE* | 95% CI | | *p* |
| --- | --- | --- | --- | --- | --- |
|  |  |  | *LL* | *UL* |  |
| Fixed effects |  |  |  |  |  |
| Intercept | 1.45x10^-15^ | .01 | -.01 | .01 | 1.00 |
| January | .02** | .01 | .01 | .03 | .007 |
| February | -.01 | .01 | -.02 | .01 | .366 |
| March | -.004 | .01 | -.02 | .01 | .520 |
| April | .01 | .01 | -.01 | .02 | .267 |
| May | .002 | .01 | -.01 | .02 | .741 |
| June | .01* | .01 | .002 | .03 | .029 |
| July | .01 | .01 | -.001 | .03 | .068 |
| August | .004 | .01 | -.01 | .02 | .577 |
| September | .01 | .01 | -.01 | .02 | .254 |
| October | -.01 | .01 | -.02 | .003 | .113 |
| November | -.02** | .01 | -.03 | -.01 | .008 |
|  | Variance | *SD* |  |  |  |
| Random effects |  |  |  |  |  |
| Individual | .43 | .66 |  |  |  |
| Residual | .57 | .75 |  |  |  |

*Note.* CI = confidence interval; *LL* = lower limit; *UL* = upper limit. Number of participants = 11,103, number of observations = 133,236. Each calendar month estimate is for the average of that month across 2012, 2013, 2014, and 2015. Each month is compared to the grand mean, i.e. time was deviation-coded.

** p* < .05. ** *p* < .01. *** *p* < .001.

**Table S3**

*Monthly green product variety sales, January to November.*

| Effect | Estimate | *SE* | 95% CI | | *p* |
| --- | --- | --- | --- | --- | --- |
|  |  |  | *LL* | *UL* |  |
| Fixed effects |  |  |  |  |  |
| Intercept | -1.84x10^-15^ | .006 | -.01 | .01 | 1.00 |
| January | -.01* | .005 | -.02 | -.001 | .035 |
| February | .01* | .005 | .001 | .02 | .026 |
| March | -.03*** | .005 | -.04 | -.02 | 9.90x10^-9^ |
| April | .01 | .005 | -.001 | .02 | .088 |
| May | -.004 | .005 | -.01 | .01 | .351 |
| June | -.001 | .005 | -.01 | .01 | .756 |
| July | .01 | .005 | -.004 | .01 | .260 |
| August | -.01** | .005 | -.02 | -.004 | .005 |
| September | .01** | .005 | .004 | .02 | .006 |
| October | .01* | .005 | .002 | .02 | .020 |
| November | .02*** | .005 | .01 | .03 | .001 |
| GnG Sales | .53*** | .003 | .52 | .53 | <2x10^-16^ |
|  | Variance | *SD* |  |  |  |
| Random effects |  |  |  |  |  |
| Individual | .40 | .63 |  |  |  |
| Residual | .25 | .50 |  |  |  |

*Note.* CI = confidence interval; *LL* = lower limit; *UL* = upper limit. Number of participants = 11,103, number of observations = 133,236. Each calendar month estimate is for the average of that month across 2012, 2013, 2014, and 2015. Each month is compared to the grand mean, i.e. time was deviation-coded.

** p* < .05. ** *p* < .01. *** *p* < .001.

**Table S4**

*Monthly sales (all products), January to November.*

| Effect | Estimate | *SE* | 95% CI | | *p* |
| --- | --- | --- | --- | --- | --- |
|  |  |  | *LL* | *UL* |  |
| Fixed effects |  |  |  |  |  |
| Intercept | 4.31x10^-14^ | .01 | -.02 | .02 | 1.00 |
| January | -.005 | .005 | -.01 | .01 | .335 |
| February | -.15*** | .005 | -.16 | -.14 | <2x10^-16^ |
| March | .07*** | .005 | .06 | .08 | <2x10^-16^ |
| April | -.10*** | .005 | -.11 | -.09 | <2x10^-16^ |
| May | .004 | .005 | -.01 | .01 | .395 |
| June | 2.70x10^-5^ | .005 | -.01 | .01 | .996 |
| July | .02*** | .005 | .01 | .03 | 8.18x10^-5^ |
| August | -.01 | .005 | -.02 | .002 | .140 |
| September | -.07*** | .005 | -.08 | -.06 | <2x10^-16^ |
| October | .01** | .005 | .01 | .02 | .003 |
| November | .07*** | .005 | .06 | .08 | <2x10^-16^ |
|  | Variance | *SD* |  |  |  |
| Random effects |  |  |  |  |  |
| Individual | .70 | .84 |  |  |  |
| Residual | .30 | .54 |  |  |  |

*Note.* CI = confidence interval; *LL* = lower limit; *UL* = upper limit. Number of participants = 11,103, number of observations = 133,236. Each calendar month estimate is for the average of that month across 2012, 2013, 2014, and 2015. Each month is compared to the grand mean, i.e. time was deviation-coded.

** p* < .05. ** *p* < .01. *** *p* < .001.

**Table S5**

*Green product sales mixed effects models with socio-demographic covariates.*

| Effect | Estimate | *SE* | 95% CI | | *p* |
| --- | --- | --- | --- | --- | --- |
|  |  |  | *LL* | *UL* |  |
| Fixed effects |  |  |  |  |  |
| Intercept | .30*** | .05 | .20 | .40 | 1.22x10^-9^ |
| January | -.01* | .005 | -.02 | -.001 | .032 |
| Green/Not Green Sales | .52*** | .003 | .52 | .53 | <2x10^-16^ |
| Env. Concern | .02** | .01 | .01 | .03 | .003 |
| Env. Concern x January | -3.31x10^-4^ | .005 | -.01 | .01 | .947 |
| Sociodemographic Variables |  |  |  |  |  |
| Age | -.001** | .001 | -.003 | .00 | .009 |
| Gender | -.21*** | .02 | -.25 | -.17 | <2x10^-16^ |
| Education | .003 | .003 | -.002 | .01 | .227 |
| Income | -.03*** | .01 | -.04 | -.02 | 3.18x10^-7^ |
| Income ∅ | -.07** | .02 | -.11 | -.02 | .002 |
| SES | .01 | .01 | -.01 | .02 | .491 |
| Occupation |  |  |  |  |  |
| Student | -.06 | .08 | -.22 | .10 | .457 |
| Homemaker | .10 | .06 | -.02 | .21 | .106 |
| Retired | .04 | .03 | -.02 | .10 | .187 |
| Carer | .03 | .07 | -.10 | .16 | .672 |
| SME | .09 | .06 | -.03 | .20 | .141 |
| Region |  |  |  |  |  |
| W. Midlands | -.06 | .03 | -.12 | .002 | .057 |
| E. Midlands | -.02 | .03 | -.08 | .04 | .551 |
| N. Ireland | -.03 | .03 | -.09 | .04 | .377 |
| S. England | -.03 | .03 | -.08 | .02 | .209 |
| E. Anglia | -.04 | .03 | -.11 | .03 | .235 |
| Scotland | -.02 | .03 | -.08 | .03 | .413 |
| Wales | -.05 | .03 | -.12 | .01 | .087 |
| N.W. England | -.01 | .03 | -.07 | .04 | .633 |
| N.E. England | -.04 | .03 | -.10 | .02 | .198 |
| London | .04 | .03 | -.02 | .11 | .214 |
| S.W. England | -.03* | .03 | -.10 | .03 | .314 |
| Region ∅ | -.02 | .02 | -.06 | .02 | .425 |
| Household Type |  |  |  |  |  |
| Own | .03 | .03 | -.03 | .09 | .311 |
| Own + Child | .004 | .04 | -.08 | .09 | .932 |
| Partner + Child | .08*** | .02 | .05 | .12 | 1.91x10^-6^ |
| Adult Family | -.01 | .02 | -.05 | .03 | .650 |
| Adult Non-Family | .11* | .05 | .02 | .20 | .018 |
| Marital Status |  |  |  |  |  |
| Single | -.003 | .03 | -.06 | .05 | .917 |
| Separated | .01 | .05 | -.08 | .11 | .783 |
| Divorced | .01 | .03 | -.05 | .07 | .771 |
| Widowed | -.05 | .04 | -.13 | .02 | .168 |
| Marital ∅ | -.13 | .09 | -.31 | .06 | .172 |
| Veg. Diet | .09*** | .02 | .05 | .13 | 4.32x10^-5^ |
|  | Variance | *SD* | *r* |  |  |
| Random effects |  |  |  |  |  |
| Individual | .32 | .57 |  |  |  |
| Slope (Env.) | .07 | .27 | .53 |  |  |
| Residual | .25 | .50 |  |  |  |

*Note.* CI = confidence interval; *LL* = lower limit; *UL* = upper limit. Number of participants = 11,103, number of observations = 133,236. Variables followed by “(∅)” refer to missing data or participants indicating they would prefer not to disclose this information. For the Gender variables, Female was chosen as the reference value.

** p* < .05. ** *p* < .01. *** *p* < .001.

**Table S6**

*Total product sales mixed effect models with socio-demographic covariates.*

| Effect | Estimate | *SE* | 95% CI | | *p* |
| --- | --- | --- | --- | --- | --- |
|  |  |  | *LL* | *UL* |  |
| Fixed effects |  |  |  |  |  |
| Intercept | -.17 | .07 | -.30 | -.04 | .013 |
| January | -.01 | .01 | -.02 | .01 | .340 |
| Env. Concern | .01 | .01 | -.01 | .02 | .465 |
| Env. Concern x January | -.01 | .01 | -.02 | .00 | .058 |
| Sociodemographic Variables |  |  |  |  |  |
| Age | -.01 | .001 | -.01 | -.01 | <2x10^-16^ |
| Gender | .18 | .03 | .12 | .23 | 5.20x10^-10^ |
| Education | 1.11x10^-4^ | .004 | -.01 | .01 | .977 |
| Income | .08 | .01 | .06 | .10 | <2x10^-16^ |
| Income ∅ | .15 | .03 | .09 | .21 | 3.84x10^-7^ |
| SES | .04 | .01 | .02 | .06 | 1.06x10^-4^ |
| Occupation |  |  |  |  |  |
| Student | -.27 | .11 | -.48 | -.06 | .011 |
| Homemaker | -.002 | .08 | -.16 | .16 | .976 |
| Retired | .09 | .04 | .01 | .18 | .024 |
| Carer | .07 | .09 | -.11 | .24 | .470 |
| SME | .09 | .08 | -.07 | .24 | .267 |
| Region |  |  |  |  |  |
| W. Midlands | -.001 | .04 | -.09 | .08 | .987 |
| E. Midlands | -.08 | .04 | -.15 | -.01 | .030 |
| N. Ireland | -.05 | .04 | -.13 | .04 | .276 |
| S. England | -.002 | .03 | -.07 | .07 | .963 |
| E. Anglia | -.02 | .05 | -.11 | .07 | .711 |
| Scotland | .01 | .04 | -.06 | .09 | .707 |
| Wales | .06 | .04 | -.02 | .15 | .138 |
| N.W. England | -.08 | .04 | -.15 | -.01 | .034 |
| N.E. England | .003 | .04 | -.08 | .09 | .941 |
| London | .02 | .04 | -.07 | .11 | .693 |
| S.W. England | -.01 | .05 | -.10 | .08 | .774 |
| Region ∅ | -.03 | .03 | -.09 | .02 | .235 |
| Household Type |  |  |  |  |  |
| Own | .00 | .04 | -.08 | .07 | .900 |
| Own + Child | .05 | .06 | -.07 | .16 | .410 |
| Partner + Child | .11 | .02 | .06 | .16 | 5.49x10^-6^ |
| Adult Family | .02 | .03 | -.04 | .08 | .480 |
| Adult Non-Family | -.09 | .06 | -.21 | .03 | .142 |
| Marital Status |  |  |  |  |  |
| Single | .05 | .04 | -.02 | .13 | .134 |
| Separated | .07 | .07 | -.06 | .20 | .279 |
| Divorced | .01 | .04 | -.08 | .09 | .901 |
| Widowed | .03 | .05 | -.06 | .13 | .495 |
| Marital ∅ | -.05 | .13 | -.30 | .20 | .679 |
| Veg. Diet | .06 | .03 | .01 | .12 | .022 |
|  | Variance | *SD* | *r* |  |  |
| Random effects |  |  |  |  |  |
| Individual | .63 | .79 |  |  |  |
| Slope (Env.) | .03 | .18 | .13 |  |  |
| Residual | .30 | .55 |  |  |  |

*Note.* CI = confidence interval; *LL* = lower limit; *UL* = upper limit. Number of participants = 11,103, number of observations = 133,236. Variables followed by “(∅)” refer to missing data or participants indicating they would prefer not to disclose this information. For the Gender variables, Female was chosen as the reference value.

** p* < .05. ** *p* < .01. *** *p* < .001.

**Table S7**

*Quarterly green product sales mixed effect model with socio-demographic covariates.*

| Effect | Estimate | *SE* | 95% CI | | *p* |
| --- | --- | --- | --- | --- | --- |
|  |  |  | *LL* | *UL* |  |
| Fixed effects |  |  |  |  |  |
| Intercept | .14*** | .03 | .10 | .19 | 9.07x10^-9^ |
| Green/Not Green Sales | .70*** | .002 | .69 | .70 | <2x10^-16^ |
| Fourth Quarter | .01 | .004 | -.003 | .01 | .213 |
| First Quarter | .01** | .004 | .003 | .02 | .009 |
| Second Quarter | .02*** | .004 | .02 | .03 | 1.37x10^-7^ |
| Env. Concern | .02*** | .01 | .01 | .04 | 7.95x10^-5^ |
| Env. Concern x Fourth Quarter | -.003 | .004 | -.01 | .01 | .488 |
| Env. Concern x First Quarter | 1.41x10^-4^ | .004 | -.01 | .01 | .975 |
| Env. Concern x Second Quarter | -.004 | .004 | -.01 | .01 | .392 |
| Sociodemographic Variables |  |  |  |  |  |
| Age | -.02** | .01 | -.04 | -.01 | .001 |
| Gender | -.08*** | .02 | -.21 | -.14 | 4.21x10^-20^ |
| Education | .01 | .01 | -.001 | .02 | .063 |
| Income | -.05*** | .01 | -.07 | -.03 | 7.18x10^-9^ |
| Income ∅ | -.07** | .02 | -.11 | -.03 | .001 |
| SES | -.001 | .01 | -.02 | .02 | .906 |
| Occupation |  |  |  |  |  |
| Student | -.04 | .07 | -.18 | .10 | .580 |
| Homemaker | .06 | .05 | -.05 | .17 | .255 |
| Retired | .02 | .03 | -.04 | .08 | .512 |
| Carer | -.03 | .06 | -.15 | .09 | .631 |
| SME | .05 | .05 | -.06 | .15 | .373 |
| Region |  |  |  |  |  |
| W. Midlands | -.06* | .03 | -.12 | -.01 | .030 |
| E. Midlands | -.02 | .03 | -.07 | .03 | .436 |
| N. Ireland | -.01 | .03 | -.07 | .05 | .775 |
| S. England | -.03 | .02 | -.08 | .02 | .203 |
| E. Anglia | -.03 | .03 | -.08 | .03 | .343 |
| Scotland | -.02 | .03 | -.08 | .03 | .361 |
| Wales | -.05 | .03 | -.11 | .001 | .056 |
| N.W. England | -.003 | .02 | -.05 | .05 | .916 |
| N.E. England | -.04 | .03 | -.10 | -.01 | .147 |
| London | .02 | .03 | -.04 | .08 | .444 |
| S.W. England | -.04 | .03 | -.10 | .02 | .183 |
| Region ∅ | -.01 | .02 | -.05 | .02 | .605 |
| Household Type |  |  |  |  |  |
| Own | .03 | .03 | -.02 | .08 | .238 |
| Own + Child | -.01 | .04 | -.09 | .06 | .724 |
| Partner + Child | .08*** | .02 | .05 | .11 | 1.66x10^-6^ |
| Adult Family | -.01 | .02 | -.05 | .03 | .722 |
| Adult Non-Family | .11* | .04 | .03 | .19 | .011 |
| Marital Status |  |  |  |  |  |
| Single | -.01 | .02 | -.06 | .04 | .636 |
| Separated | .02 | .04 | -.07 | .10 | .738 |
| Divorced | .02 | .03 | -.03 | .08 | .406 |
| Widowed | -.05 | .03 | -.12 | .02 | .133 |
| Marital ∅ | -.11 | .09 | -.28 | .06 | .191 |
| Veg. Diet | .10*** | .02 | .06 | .14 | 8.64x10^-8^ |
|  | Variance | *SD* |  |  |  |
| Random effects |  |  |  |  |  |
| Individual | .28 | .53 |  |  |  |
| Residual | .34 | .58 |  |  |  |

*Note.* CI = confidence interval; *LL* = lower limit; *UL* = upper limit. Number of participants = 11,103, number of observations = 133,236. Variables followed by “(∅)” refer to missing data or participants indicating they would prefer not to disclose this information. For the Gender variables, Female was chosen as the reference value.

** p* < .05. ** *p* < .01. *** *p* < .001.

**Table S8**

*Quarterly total product sales mixed effect model with socio-demographic covariates.*

| Effect | Estimate | *SE* | 95% CI | | *p* |
| --- | --- | --- | --- | --- | --- |
|  |  |  | *LL* | *UL* |  |
| Fixed effects |  |  |  |  |  |
| Intercept | -.20*** | .04 | -.27 | -.13 | 1.53x10^-8^ |
| Fourth Quarter | .08*** | .005 | .07 | .09 | <2x10^-16^ |
| First Quarter | .01* | .005 | .002 | .02 | .018 |
| Second Quarter | .01 | .005 | -.003 | .02 | .150 |
| Env. Concern | .01 | .01 | -.01 | .02 | .344 |
| Env. Concern x Fourth Quarter | -.01* | .005 | -.02 | -.001 | .034 |
| Env. Concern x First Quarter | -.004 | .005 | -.01 | .01 | 463 |
| Env. Concern x Second Quarter | -.003 | .005 | -.01 | .01 | .564 |
| Sociodemographic Variables |  |  |  |  |  |
| Age | -.09*** | .01 | -.11 | -.07 | 1.74x10^-17^ |
| Gender | .17*** | .03 | .12 | .22 | 3.04x10^-10^ |
| Education | 1.16x10^-4^ | .01 | -.02 | .02 | .989 |
| Income | .11*** | .01 | .09 | .14 | 1.97x10^-20^ |
| Income ∅ | .15*** | .03 | .09 | .20 | 3.18x10^-7^ |
| SES | .05*** | .01 | .03 | .08 | 1.26x10^-4^ |
| Occupation |  |  |  |  |  |
| Student | -.25* | .10 | -.44 | -.05 | .014 |
| Homemaker | -.01 | .08 | -.16 | .14 | .874 |
| Retired | .09* | .04 | .01 | .17 | .021 |
| Carer | .05 | .09 | -.12 | .21 | .583 |
| SME | .08 | .08 | -.07 | .23 | .301 |
| Region |  |  |  |  |  |
| W. Midlands | .004 | .04 | -.08 | .08 | .919 |
| E. Midlands | -.08* | .04 | -.15 | -.01 | .030 |
| N. Ireland | -.04 | .04 | -.12 | .04 | .309 |
| S. England | -.01 | .03 | -.07 | 06 | .876 |
| E. Anglia | -.01 | .04 | -.10 | .07 | .785 |
| Scotland | .01 | 04 | -.06 | .08 | .765 |
| Wales | .06 | .04 | -.02 | .14 | .146 |
| N.W. England | -.08* | .03 | -.14 | -.01 | .025 |
| N.E. England | .002 | .04 | -.08 | .08 | .952 |
| London | .01 | .04 | -.07 | .10 | .788 |
| S.W. England | -.01 | .04 | -.10 | .07 | .760 |
| Region ∅ | -.04 | .03 | -.09 | .01 | .115 |
| Household Type |  |  |  |  |  |
| Own | -.01 | .04 | -.08 | .07 | .859 |
| Own + Child | .04 | .05 | -.07 | .14 | .493 |
| Partner + Child | .10*** | .02 | .06 | .15 | 4.42x10^-6^ |
| Adult Family | .02 | .03 | -.03 | .07 | .468 |
| Adult Non-Family | -.07 | .06 | -.19 | .04 | .206 |
| Marital Status |  |  |  |  |  |
| Single | .05 | .03 | -.02 | .11 | .174 |
| Separated | .08 | .06 | -.05 | .20 | .210 |
| Divorced | .01 | .04 | -.07 | .08 | .875 |
| Widowed | .04 | .05 | -.06 | .13 | .442 |
| Marital ∅ | -.06 | .12 | -.29 | .18 | .621 |
| Veg. Diet | .06* | .03 | .01 | .12 | .014 |
|  | Variance | *SD* |  |  |  |
| Random effects |  |  |  |  |  |
| Individual | .57 | .76 |  |  |  |
| Residual | .41 | .64 |  |  |  |

*Note.* CI = confidence interval; *LL* = lower limit; *UL* = upper limit. Number of participants = 11,103, number of observations = 133,236. Variables followed by “(∅)” refer to missing data or participants indicating they would prefer not to disclose this information. For the Gender variables, Female was chosen as the reference value.

** p* < .05. ** *p* < .01. *** *p* < .001.
