# Supplementary material for: New year as a moment of change in pro-environmental product consumption: evaluating the habit discontinuity and self-activation hypotheses using a large UK retail dataset
Source: Front Psychol. 2025 Apr 23;16:1550091. doi: 10.3389/fpsyg.2025.1550091 (PMC12055770; doi:10.3389/fpsyg.2025.1550091)
Supplement: Supplementary file 2 [file Table_1.docx]

**Appendix**

**Table A1**

*List of variables in tables/models and how they are measured*

| Variable in Tables | Full Variable Name | Variable Measurement |
| --- | --- | --- |
| GnG sales | Green/Not Green Sales | Green product sales were measured within 31 different categories (types) of product, categories where both green products and non-green products were available. GnG sales is the count of all these sales (green and non-green) at the monthly level for individuals. |
| Env. Concern | Environmental Concern | Items rated on a 7-point Likert scale (1= strongly disagree, 7 = strongly agree):   - Environmental considerations affect the products that I purchase - I am concerned about climate change (also known as global warming) |
| Age | Age | Age, in years, self-reported on continuous scale |
| Gender | Gender | Self-reported gender, where responses were coded as follows:  “1” = Responded “Female”  “0” = Responded “Male”  Hence, Male was chosen as the reference value. |
| Education | Highest Level of Education | Responded 1-11 to question: “What is your highest level of education?”   1. Completed some secondary school 2. GCSE(s) or equivalent 3. BTEC or equivalent 4. AS-level 5. A-level or equivalent 6. Bachelor’s degree 7. Other postgraduate qualification (e.g., postgraduate diploma) 8. Master’s degree 9. PhD, law, or medical degree 10. Other advanced degree beyond a master’s 11. Other – please specify   AS, A-level and BTEC were each recoded “3” and other advanced degree “9”. “Other” responses were recoded or omitted according to specification. |
| Income | Total Annual Income | Responded 1-8 to question: “What is your total annual household income before tax?”   1. Less than £25,000 2. £25,000 to £34,999 3. £35,000 to £49,999 4. £50,000 to £74,999 5. £75,000 to £99,999 6. £100,000 to £149,999 7. £150,000 or more 8. Prefer not to say   Responses were coded thus:   - 1” = Responded 1 - “2” = Responded 2 - “3” = Responded 3 - “4” = Responded 4 or 5 - “5” = Responded 6 or 7 - “Income ∅“ = Responded 8 or missing data |
| Income ∅ | Total Annual Income (Null) |  |
| SES | Occupational Socioeconomic Status | A scale from 1-4 based on responses to the question: “Which of the following best describes the occupation of the main income earner in your household?”   1. Semi or unskilled manual work e.g. Manual workers, all apprentices to be skilled trades, Caretaker, Park Keeper, non-HGV driver, Shop Assistant 2. Skilled manual worker e.g. Skilled Bricklayer, Carpenter, Plumber, Painter, Bus/ Ambulance Driver, HGV Driver, AA Patrolman, Pub/Bar Worker etc. 3. Supervisory or clerical/ junior managerial/ professional/ administrative e.g. Office worker, Student Doctor, Foreman with 25+ employees, Salesperson, etc. 4. Intermediate managerial/professional/administrative e.g. Newly qualified (under 3 years) Doctor, Solicitor, Board Director small organisation,  Middle Manager in large organisation, Principle Officer in Civil Service/Local Government 5. Higher managerial/professional/administrative e.g. Established Doctor, Solicitor, Board Director in a large organisation (200+ employees), top level Civil Servant/Public Service Employee 6. Student 7. Casual worker - not in permanent employment 8. Housewife/ Homemaker 9. Retired and living on State Pension 10. Unemployed or not working due to long-term sickness 11. Full-time carer of other household member 12. Other – please specify   Responses were coded as following:   - “1” = Responded 1, 7, or 10 - “2” = Responded 2 - “3” = Responded 3 - “4” = Responded 4 or 5 |
| Occupation |  | Based on responses to the question: “Which of the following best describes the occupation of the main income earner in your household?”   1. Semi or unskilled manual work e.g. Manual workers, all apprentices to be skilled trades, Caretaker, Park Keeper, non-HGV driver, Shop Assistant 2. Skilled manual worker e.g. Skilled Bricklayer, Carpenter, Plumber, Painter, Bus/ Ambulance Driver, HGV Driver, AA Patrolman, Pub/Bar Worker etc. 3. Supervisory or clerical / junior managerial / professional/ administrative e.g. Office worker, Student Doctor, Foreman with 25+ employees, Salesperson, etc. 4. Intermediate managerial/professional/administrative e.g. Newly qualified (under 3 years) Doctor, Solicitor, Board Director small organisation,  Middle Manager in large organisation, Principle Officer in Civil Service/Local Government 5. Higher managerial / professional / administrative e.g. Established Doctor, Solicitor, Board Director in a large organisation (200+ employees), top level Civil Servant/Public Service Employee 6. Student 7. Casual worker - not in permanent employment 8. Housewife/Homemaker 9. Retired and living on State Pension 10. Unemployed or not working due to long-term sickness 11. Full-time carer of other household member 12. Other – please specify   Dummy variables created based on the following responses:   - “Student” = Responded 6 - “Homemaker” = Responded 8 - “Retired” = Responded 9 - “Carer” = Responded 11   “SME” reflected “other” answers indicating self-employment or running one’s own business. ) |
| Student | Student |  |
| Homemaker | Homemaker |  |
| Retired | Retired |  |
| Carer | Carer |  |
| SME | Small- and Medium-sized Enterprise (SME) Owner |  |
| Region |  | The survey asked the respondents to choose the region where they live from a list of regions. Answers were dummy-coded by region, with Yorkshire as reference category. |
| W. Midlands | West Midlands |  |
| E. Midlands | East Midlands |  |
| N. Ireland | Northern Ireland |  |
| S. England | South England |  |
| E. Anglia | East Anglia |  |
| Scotland | Scotland |  |
| Wales | Wales |  |
| N.W. England | North West England |  |
| N.E. England | North East England |  |
| London | London |  |
| S.W. England | South West England |  |
| Yorkshire | Yorkshire |  |
| Region ∅ | Region (Null) | Missing data, or indicated preference to not disclose this information. |
| Household Type |  | Based on responses to the question: “Please could you confirm which household situation best applies to you?”   1. Living on my own (no children or children have left home) 2. Living on my own with children under 18 3. Living with partner/spouse (no children or children have left home) 4. Living with partner/spouse with children under 18 5. Living with other adult family members (i.e., aged 18 or older) e.g., adult children, parents, and/or elderly relatives 6. Living with other adults that are non-family members e.g., friends, flatmates   Dummy variables created based on the following responses:   - “Own” = Responded 1 - “Own + Child” = Responded 2 - “Partner + Child” = Responded 4 - “Adult Family” = Responded 5 - “Adult Non-Family” = Responded 6   Response 3 was selected as a reference value. |
| Own | Living Alone |  |
| Own + Child | Living Alone with Child |  |
| Partner + Child | Living Alone with Partner and Child |  |
| Adult Family | Living with Adult Family Member(s) |  |
| Adult Non-Family | Living with Non-Family Adult(s) |  |
| Marital Status |  | Based on responses to the question: “What is your marital status?”   1. Single, never married 2. Married, civil or domestic partnership/living with a partner 3. Widowed 4. Divorced 5. Separated 6. Other – please specify   Dummy variables created based on the following responses:   - “Single” = Responded 1 - “Separated” = Responded 5 - “Divorced” = Responded 4 - “Widowed” = Responded 3 - “Marital ∅” = missing data   Response 2 was selected as a reference value. |
| Single | Single |  |
| Separated | Separated |  |
| Divorced | Divorced |  |
| Widowed | Widowed |  |
| Marital ∅ | Marital (Null) |  |
| Veg. Diet | Vegetarian or Vegan Diet | Based on responses to the question: “Do you have any specific dietary requirements?”   1. Vegetarian 2. Vegan 3. Pescetarian 4. Diabetic 5. Gluten intolerant 6. Kosher 7. Lactose intolerant 8. Allergic to nuts 9. Allergic to fish 10. None 11. Other – please specify   Binary variable was created based on the following responses:   - “Veg. Diet” = Responded 1 or 2 |
